# Evolution of Large Polymorphic Inversions in a Panmictic Songbird

**DOI:** 10.1093/molbev/msaf262

**Published:** 2025-10-16

**Authors:** Yifan Pei, Wolfgang Forstmeier, Alexander Suh, Lynn Bambach, Inês Borges, Gabriel Weijie Low, Anne-Marie Dion-Côté, Ulrich Knief, Jochen Wolf, Bart Kempenaers

**Affiliations:** Department of Organismal Biology—Systematic Biology, Evolutionary Biology Centre, Science for Life Laboratory, Uppsala University, Norbyv. 18D, Uppsala 75236, Sweden; Centre for Molecular Biodiversity Research, Leibniz Institute for the Analysis of Biodiversity Change, Adenauerallee 127, Bonn 53113, Germany; Department of Ornithology, Max Planck Institute for Biological Intelligence, Eberhard-Gwinner-Str. 7, Seewiesen 82319, Germany; Institute of Evolutionary Biology and Ecology, University of Bonn, An der Immenburg 1, Bonn 53121, Germany; Department of Ornithology, Max Planck Institute for Biological Intelligence, Eberhard-Gwinner-Str. 7, Seewiesen 82319, Germany; Department of Organismal Biology—Systematic Biology, Evolutionary Biology Centre, Science for Life Laboratory, Uppsala University, Norbyv. 18D, Uppsala 75236, Sweden; Centre for Molecular Biodiversity Research, Leibniz Institute for the Analysis of Biodiversity Change, Adenauerallee 127, Bonn 53113, Germany; Institute of Evolutionary Biology and Ecology, University of Bonn, An der Immenburg 1, Bonn 53121, Germany; Faculty of Mathematics, Informatics, and Technology—RheinAhrCampus Remagen, University of Applied Sciences Koblenz, Joseph-Rovan-Allee 2, Remagen 53424, Germany; Centre for Molecular Biodiversity Research, Leibniz Institute for the Analysis of Biodiversity Change, Adenauerallee 127, Bonn 53113, Germany; Institute of Evolutionary Biology and Ecology, University of Bonn, An der Immenburg 1, Bonn 53121, Germany; Evolution of Sensory and Physiological Systems Department, Max Planck Institute for Biological Intelligence, Eberhard-Gwinner-Str. 5, Seewiesen 82319, Germany; National Parks Board, Singapore Botanical Gardens, 1 Cluny Road, Singapore 259569, Singapore; Département de Biologie, Université de Moncton, 18, av. Antonine-Maillet, Moncton, NB, E1A 3E9, Canada; Evolutionary Biology and Ecology, Faculty of Biology, University of Freiburg, Hauptstr. 1, Freiburg 79104, Germany; Division of Evolutionary Biology, Faculty of Biology, LMU Munich, Großhaderner Straße 2, Planegg-Martinsried D-82152, Germany; Department of Microevolution and Biodiversity, Max Planck Institute for Biological Intelligence, Eberhard-Gwinner-Str. 7, Seewiesen 82319, Germany; Department of Ornithology, Max Planck Institute for Biological Intelligence, Eberhard-Gwinner-Str. 7, Seewiesen 82319, Germany

**Keywords:** microchromosome, inversion polymorphism, heterosis, linked-read sequencing, infertility, fecundity, offspring survival, lifespan, *Taeniopygia guttata*

## Abstract

Chromosomal inversions have long been appreciated as an important source of genetic diversity, local adaptation, and speciation. However, selection pressures maintaining ancestral and derived alleles at high frequency over extended periods of time remain poorly characterized. Using genome-wide single-nucleotide polymorphism markers and shared barcodes of linked-read sequences from 20 wild and 7 captive zebra finches *Taeniopygia guttata*, we systematically scanned a high-quality zebra finch reference genome and identified all large polymorphic inversions that segregate at high minor allele frequencies. Apart from the known polymorphic inversions on chromosomes *Tgu5*, *Tug11*, *Tgu13*, and *TguZ*, we characterized two inversions on microchromosomes *Tgu26* and *Tgu27* and identified another eight putative inversions, located mostly on microchromosomes and ranging in size from 0.42 to 65.22 Mb. Population genomic analyses show that most of the six *bona fide* inversions are complex, containing short nested inversions. The early inversions emerged an estimated 0.6 to 2.2 million years ago and segregate at relatively high frequencies in the wild (minor haplotype frequency range: 0.289 to 0.429). Based on fitness-related measures of about 5,000 captive zebra finches, we conclude that three of the inversion polymorphisms (*Tgu11*, *Tgu27*, and *TguZ*) may be maintained by net heterosis. In the youngest of the six inversions (*Tgu13*), the derived haplotype showed weak positive additive effects on various fitness components. In combination with previous discoveries, we provide a comprehensive overview of the genomic distribution and evolutionary dynamics of large polymorphic inversions in the panmictic zebra finch. Our findings highlight (i) that microchromosomes may harbor quite a few additional inversion polymorphisms, (ii) that most of the inversions contain smaller nested or overlapping inversions, and (iii) that inversions were most likely maintained by weak heterosis with small fitness effects requiring large sample sizes to be detected.

## Introduction

Chromosomal inversions physically link hundreds of genes and have frequently been associated with drastic phenotypic differences in morphology and behavior (supergenes), e.g. in birds (Küpper et al. 2015; Lamichhaney et al. 2015; Tuttle et al. 2016; Sanchez-Donoso et al. 2022), mammals (Hager et al. 2022), insects (Le Poul et al. 2014; Pracana et al. 2017; Jay et al. 2018; Mérot et al. 2020), and plants (Lowry and Willis 2010; Lee et al. 2016). While the study of the genetic basis of striking phenotypic polymorphisms has led to the discovery of many supergenes (i.e. top-down or forward approach), an increasing number of inversion polymorphisms across the tree of life have also been detected either cytogenetically (e.g. through shifted centromere positions; Itoh and Arnold 2005; Itoh et al. 2011) or bioinformatically (using panels of single-nucleotide polymorphisms [SNPs] that form large linkage blocks, e.g. Knief et al. 2016; Harringmeyer and Hoekstra 2022, or using long-read sequencing or Hi-C, e.g. Harewood et al. 2017; Geng et al. 2023). In these cases of phenotype-independent discovery, the phenotypic effects, fitness consequences, and maintenance mechanisms of the polymorphism typically remain unclear. Reduced recombination between the derived inversion and ancestral haplotypes leads to independent evolution of large genomic regions, which may eventually promote reproductive isolation and speciation (Hoffmann et al. 2004; Kirkpatrick and Barton 2006; Faria et al. 2019; Knief et al. 2024). Despite being ubiquitous and of evolutionary importance, our current knowledge about the evolution of inversions is biased toward classical model species such as flies (Hoffmann et al. 2004) and toward cases where the inversions show striking phenotypic effects. This bias may largely be due to the fact that the detection of inversion polymorphisms is non-trivial and quantification of their fitness consequences can be demanding. To better understand the evolutionary significance of polymorphic inversions, comprehensive empirical studies are needed (Faria et al. 2019).

The Australian zebra finch *Taeniopygia guttata castanotis* is a well-studied songbird species that is native to and widely distributed across Australia (Zann 1996). Its high-quality reference genome consists of 40 chromosomes which have been classified into seven macrochromosomes *Tgu1* to *Tgu5*, *Tgu1A*, and *TguZ* (62 to 152 Mb), seven intermediate chromosomes *Tgu6* to *Tgu12* (20 to 40 Mb), and 26 microchromosomes (1 to 20 Mb) (Fillon 1998) based on cytogenetic resolution (Waters et al. 2021). Zebra finches have at least four large (12 to 63 Mb) inversion polymorphisms that segregate at an allele frequency of ∼50% in the wild and are located on chromosomes *Tgu5*, *Tgu11*, *Tgu13*, and *TguZ* (Itoh and Arnold 2005; Itoh et al. 2011; Knief et al. 2016). Based on data of multiple phenotypic and life-history traits of a zebra finch population, earlier studies established that two of these inversions are likely maintained by net heterosis, whereby heterokaryotypic individuals exhibit overall higher fitness than homokaryotypic genotypes. For the *TguZ* inversion, heterokaryotypic males showed increased sperm motility and hence siring success (Kim et al. 2017; Knief et al. 2017a, 2021), while for the *Tgu11* inversion, the derived allele appeared to additively increase female fecundity and male siring success, but to reduce individual survival in the homozygous state (Pei et al. 2023).

A previous scan for polymorphic inversions (Knief et al. 2016) had insufficient power to detect inversions on the majority of the microchromosomes (in particular those 19 that are <10 Mb in length) (Warren et al. 2010; Rhie et al. 2021). Patterns of linkage disequilibrium (LD) suggested an inversion on microchromosomes *Tgu26* and *Tgu27*, but a lack of informative SNP markers precluded further investigation (Knief et al. 2016). Moreover, at the time of the previous study, six microchromosomes (*Tgu29* to *Tgu34*) had not yet been assembled (Warren et al. 2010).

Here, we aim (i) to identify all large inversion polymorphisms in the zebra finch genome (pragmatically defined as spanning at least 3 Mb on macrochromosomes and intermediate chromosomes and 0.4 Mb on microchromosomes) and (ii) to understand the selective forces shaping frequencies of ancestral and derived alleles segregating in the wild. To this end, we used conventional Illumina and 10X linked-read sequencing data of wild and captive individuals that were published in Singhal et al. (2015), Kinsella et al. (2019), Rhie et al. (2021), and Pei et al. (2022). First, we used genome-wide SNP data from 19 wild adults that were captured in southeastern Australia (Singhal et al. 2015) to screen all chromosomes for large structural polymorphisms using principal component analysis (PCA) and to perform population genomic analysis. We then verified the detected inversions using 10X linked-read sequencing data from eight additional individuals (Kinsella et al. 2019; Rhie et al. 2021; Pei et al. 2022). We identified the ancestral arrangements and determined their divergence time using multisequence alignments of the 19 zebra finches and multispecies alignments of the zebra finch assembly with 13 closely related species that have publicly available chromosome-level assemblies. Lastly, we estimated the fitness effects of these inversions using long-term fitness data obtained from about 5,000 birds breeding in captivity to study the evolutionary mechanisms that can maintain these polymorphisms. We discuss how these polymorphisms might have been maintained over time.

## Results

### Detection and Characterization of Inversion Polymorphisms

**Fig. 1.**
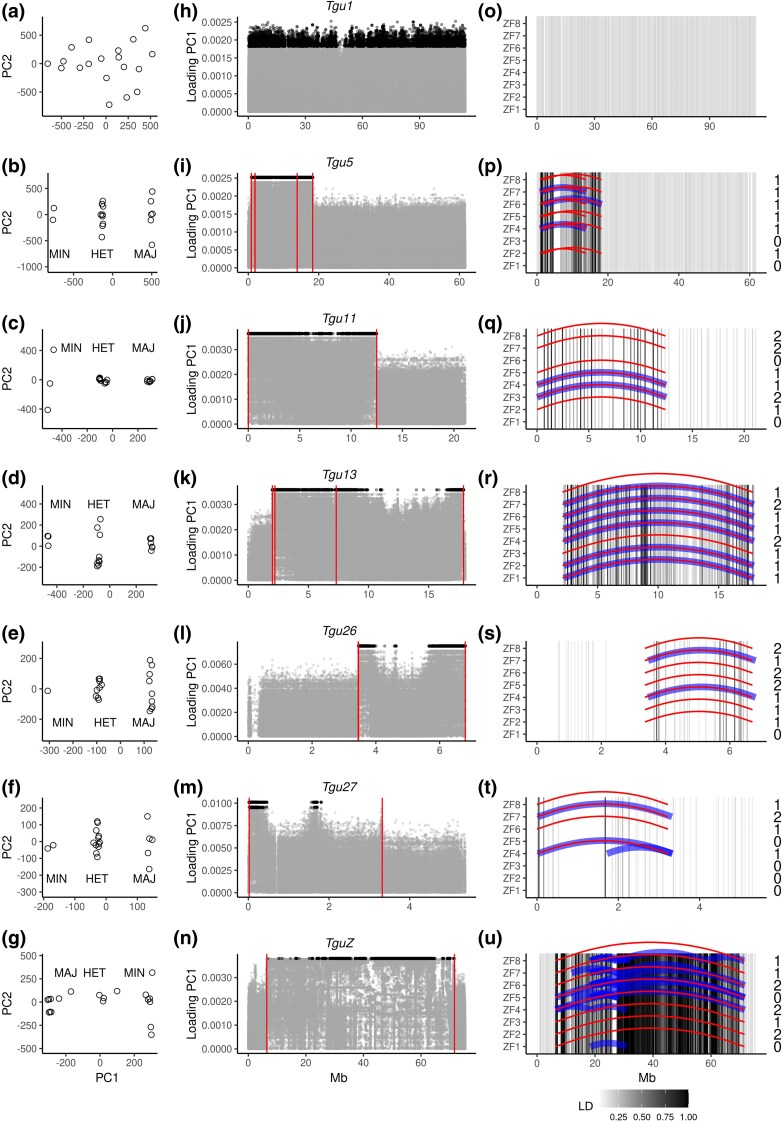
Characterization of six large polymorphic inversions in the zebra finch. The left column a to g) shows the scores of PC1 and PC2 of chromosome-wide SNPs among 19 wild zebra finches (open circles). Individuals homozygous for the major (Maj) or the minor (Min) allele and heterozygous (Het) individuals were defined based on clusters of individuals along PC1. Major and minor alleles were defined based on the allele frequencies in the 19 wild birds. The middle column h to n) shows the absolute loadings of SNPs (gray and black dots) on PC1 along chromosomes. Black dots are the top 5% SNPs with the highest loading on PC1 within each chromosome. The right column o to u) displays the distribution of unexpected intra-chromosomal barcode-sharing over long distances (blue and red curved lines) in eight sequenced individuals (rows) relative to the reference assembly (20). Blue lines link high-quality calls of intra-chromosomal barcode interactions detected by Long Ranger that are more than 3 Mb apart and that were found in more than one individual (for outlier calls from single individuals, see Figure S3). Red lines depict manually validated interactions based on raw barcode interaction plots (blindly called from Figure S4) in chromosomes that showed a signal of an inversion in the PCA. Numbers indicate the expected number of alternative inversion types (relative to the reference; 0 = same as reference). Each vertical bar is a SNP that was used in Knief et al. (2016). SNP positions were lifted from the old assembly TaeGut1 (Warren et al. 2010) to the assembly used in this study (Rhie et al. 2021). The shading of each bar (gray to black) indicates the highest level of LD of this SNP with another SNP that is at least 1 Mb apart within the same chromosome, among 948 wild zebra finches (Knief et al. 2016). The first row shows a negative example of chromosome *Tgu1* without an inversion polymorphism. The large blocks of SNPs on chromosomes *Tgu5*, *Tgu11*, *Tgu13*, and *TguZ* that are in high LD (black bars) indicate inversions. The microchromosomes *Tgu26* and *Tgu27* showed only weak signals of LD suggesting the presence of inversions (Knief et al. 2016). Note that all high-quality distant barcode interactions that were detected by Long Ranger were on chromosomes that contained inversions (*N* = 39 blue lines on the six chromosomes that show a signal of segregating inversions, i.e. *Tgu5*, *Tgu11*, *Tgu13*, *TguZ*, *Tgu26*, and *Tgu27*). Also note that *TguZ* contains many nested inversions.

PCA of SNPs from 19 wild zebra finches (W1 to W19 in Table S1) revealed the typical pattern of inversion polymorphisms with two major haplotypes in the four chromosomes known to contain an inversion polymorphism (*Tgu5*, *Tgu11*, *Tgu13*, and *TguZ*; Itoh and Arnold 2005; Itoh et al. 2011 ; Knief et al. 2016), as well as in an additional 10 chromosomes (*Tgu7*, *Tgu8*, *Tgu26* to *Tgu31*, *Tgu33*, and *Tgu34*; Fig. 1; Figure S1). The 14 putative inversions vary in size from 0.42 to 65.22 Mb and have a minor allele frequency ranging from 0.184 to 0.500 (Tables S2 and S3). Among these 14 putative inversions, the breakpoints on chromosomes *Tgu5*, *Tgu11*, *Tgu13*, *Tgu26*, *Tgu27*, and *TguZ* could be verified in the linked-read sequencing data (Fig. 1) and from tag SNPs that had been designed from 948 previously genotyped wild birds (Knief et al. 2016). We focused on these six inversion polymorphisms in the following analyses.

For each of the six chromosomes, the 19 wild zebra finches grouped into three clusters along PC1, with presumed heterokaryotypic individuals falling into the central cluster, and the two types of homozygotes on either side (Fig. 1b to g). The allele frequencies of these inversion haplotypes matched with previous work (Knief et al. 2016) (Table 1; Figure S2). Minor allele frequencies ranged from 0.29 to 0.50 (note that *TguZ* contains two minor alleles B and C; Knief et al. 2016) and allele frequencies showed no sign of Hardy–Weinberg disequilibrium (all *P* > 0.2; Fig. 1; Table S2). On all six chromosomes, the SNPs with the highest (top 0.5%) absolute value of loading on PC1 were detected as a large block, with the absolute loading gradually decreasing toward the center of the block (Fig. 1i to n). We expect that the physical position of the first and last of these SNPs pinpoints the inversion breakpoints (Fig. 1i to n).

**Table 1:** Allele frequency, age, and ancestral state of the six large inversions segregating in the zebra finch

Chromosome	Genotype (948 wzf in Knief et al. (2016))	Genotype (19 wzf)	Allele frequency (948 wzf in Knief et al. (2016))	Allele frequency (19 wzf)	Ancestral state	Divergence time in million years with its 95% HPD (10 kb auto and 100 kb Z)
5	AA	Major	0.595	0.605	Ancestral	1.4 [1.025 to 1.698]
5	BB	Minor	0.405	0.395	Derived	
11	AA	Major	0.526	0.605	Derived	1.2 [0.714 to 1.627]
11	BB	Minor	0.474	0.395	Ancestral	
13	AA	Major	0.525	0.605	Derived	0.6 [0.389 to 0.745]
13	BB	Minor	0.475	0.395	Ancestral	
26	AA	Major	…	0.711	Derived	1.8 [1.374 to 2.161]
26	BB	Minor	…	0.289	Ancestral	
27	AA	Major	…	0.579	Ancestral	1.2 [0.668 to 1.636]
27	BB	Minor	…	0.421	Derived	
Z	AA	Major	0.596	0.500	Derived	2.2^a^ [1.596 to 2.911]
Z	BB	Minor	0.330	0.429	Derived from C	
Z	CC	Minor	0.074	0.071	Derived from A	

Following Knief et al. (2016), the major haplotype is referred to as “A” and the minor haplotype as “B” based on their allele frequencies among 948 wild zebra finches (wzf; also see Figure S2).

HPD, highest posterior density.

^a^Z divergence time estimation was performed by combining homozygous AA males with AW females and contrasting them against a combination of homozygous BB males and BW females. Note that *TguZ* contains two minor alleles B and C (Knief et al. 2016); however, among the 19 wild zebra finches used in this study, no homozygous CC males and no CW females were observed.

To further verify the location and to characterize the breakpoints of the inversions, we used barcode-sharing information derived from linked-read sequencing of eight additional zebra finches (ZF1 to ZF8 in Table S1). The putative inversion types of these eight individuals on all inverted chromosomes were predicted from the SNP loadings of the PCA conducted on the 19 wild zebra finches (Fig. 1; also see Figure S2; for details, see Materials and Methods). Long-range (≥0.4 Mb in microchromosomes smaller than 20 Mb and ≥3 Mb in chromosomes larger than 20 Mb) intra-chromosomal barcode-sharing was almost exclusively observed on the aforementioned six inverted chromosomes and only in those individuals carrying at least one different arrangement than the reference assembly (Fig. 1; Figure S3). Note that the calls from other chromosomes (Figure S3) may represent a mix of true inversions with a low minor allele frequency, false positives due to duplications of long repetitive sequences in the genome, or mis-assemblies in the reference. In all cases, the putative inversion breakpoints identified by intra-chromosomal barcode-sharing on the six inverted chromosomes (Fig. 1p to u) coincided with the regions of high SNP loadings on PC1 (Fig. 1i to n). The combined evidence of these two independent approaches identifies these six cases as *bona fide* inversions. And the inversion breakpoints on chromosomes *Tgu5*, *Tgu11*, *Tgu13*, *Tgu26*, and *Tgu27* were mapped with an average resolution of 8 kb (range: 43 to 58,094 bp) on the reference assembly (Fig. 1i to n and p to u; Table S3; also see Figure S4).

Four out of the six *bona fide* inversions appeared to contain either smaller overlapping or nested inversions. On chromosomes *Tgu11* and *Tgu26*, there was only a single pair of distant barcode interactions, suggesting that the current arrangement resulted from a single structural mutation. Using less stringent detection criteria (allowing calls close to an assembly gap in the case of *Tgu13* and single occurrence in the case of *Tgu27*) revealed a second, smaller inversion within the major inverted haplotype in chromosomes *Tgu13* and *Tgu27* (Figures S3 and S4; note that for *Tgu13*, this is consistent with PCA using 948 birds where AA individuals further group into at least three clusters and AB individuals group into at least two clusters along PC2) (Knief et al. 2016; Figure S2c). Chromosome *Tgu5* contained two overlapping inversions on the same haplotype (Fig. 1p). The sex chromosome *TguZ* had the largest number of unexpected distant barcode interactions (Fig. 1u), suggesting that it contained many nested or overlapping small inversions. Because individuals did not cluster along PC2, we cannot infer their genotypes for the smaller (nested or overlapping) inversions. Therefore, in the following analyses, we focused on the two largest inversion haplotypes for each of the six chromosomes separated along PC1 in Fig. 1.

In summary, for the six *bona fide* inversions, the locations of the breakpoints that were detected through PCA and barcode-sharing overlapped and coincided with those mapped at lower spatial resolution among 948 wild-caught zebra finches (Knief et al. 2016) (Fig. 1p to u). Although the actual breakpoints on chromosome *TguZ* could be missing in the current genome assembly, we also treated the most distant pair of reads with shared barcodes on *TguZ* as putative breakpoints for the following ancestral-type and population-genetic analyses.

### Ancestry Reconstruction

**Fig. 2.**
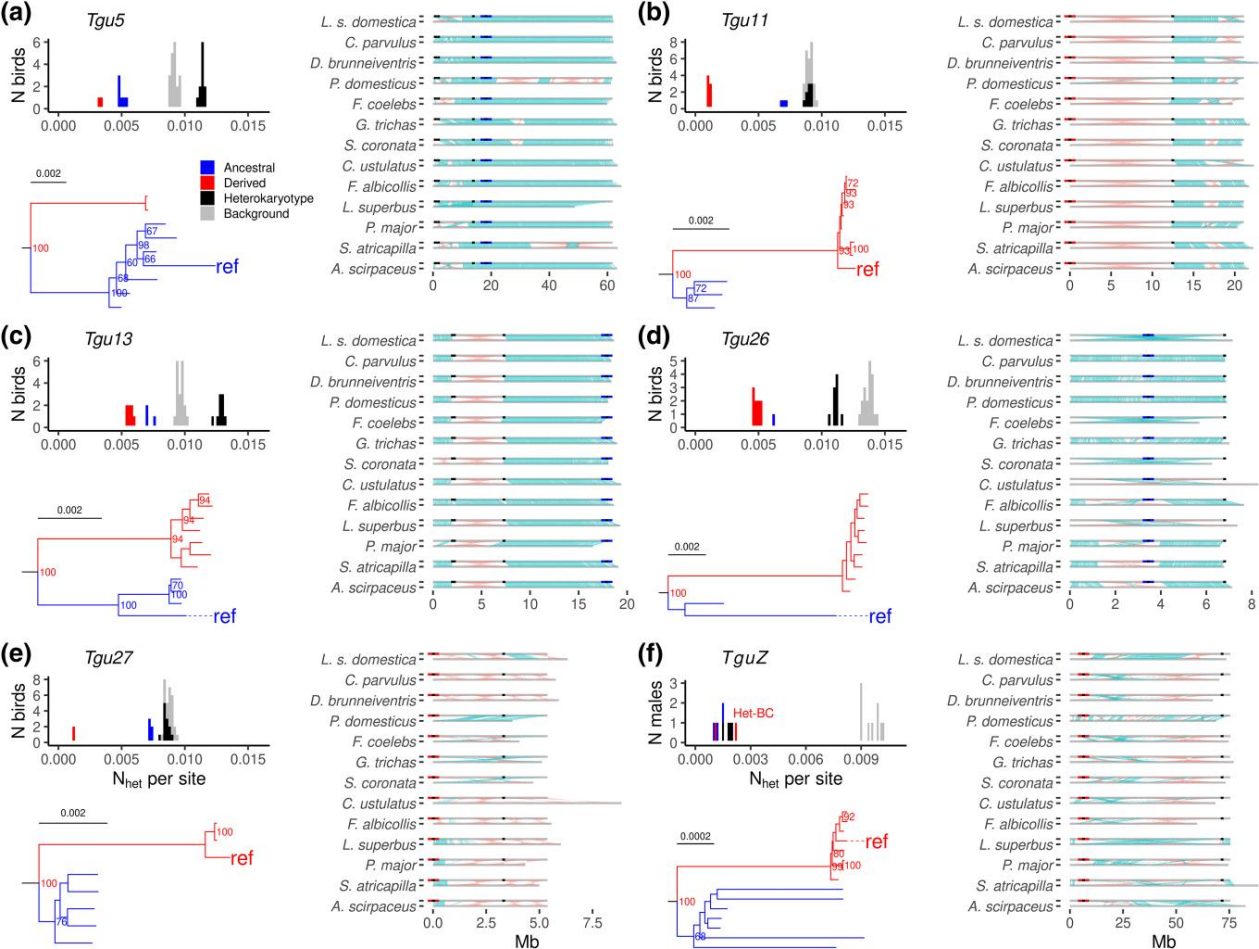
Ancestral-state analysis of six zebra finch inversions. Histograms of heterozygosity and phylogenetic trees in relation to an individual's inversion type (predicted via PCA in Fig. 1), as well as pairwise alignments between the zebra finch reference assembly and 13 songbird assemblies for inversions on chromosomes *Tgu5* a), *Tgu11* b), *Tgu13* c), *Tgu26* d), *Tgu27* e), and *TguZ* f). Blue depicts individuals that are homozygous for the ancestral allele (or the less derived for *TguZ*), red depicts individuals that are homozygous for the more derived allele, and black indicates heterokaryotypic individuals. Both alleles in *TguZ* f) are likely derived; therefore, here blue indicates the relatively older haplotype. Het-BC f) indicates an individual that is heterokaryotypic for inversion types B and C on *TguZ*. In histograms of each chromosome, for each of the 19 wild zebra finches, the number of heterozygous SNPs (N_het_) per base pair was calculated for sites within 10% of the length in the inverted region adjacent to either breakpoint (red, blue, and black) and outside of the inverted region as background (gray). Note that individuals homozygous for the derived inversion type show a lower number of heterozygous SNPs per site compared to the homozygous ancestral ones. Phylogenetic trees were built using a 400 kb sequence adjacent to one of the breakpoints (selected sequences are indicated as blue and red lines in the multispecies alignments). Here, we constructed one sequence per individual to build trees, using IUPAC codes to indicate heterozygous sites. Trees were rooted using the double-barred and long-tailed finch (not shown). Bootstrap support values are shown if larger than 60. Scale bars show the number of substitutions per site. The right-side panels for each chromosome show 13 pairwise alignments between the zebra finch reference assembly and each of the 13 songbird assemblies. In each pairwise alignment, the top line (unlabeled) represents the zebra finch reference, while the bottom line (labeled with species name) corresponds to one of the other songbird species. Light blue shows aligned sequence collinearity whereas pink indicates reversed sequence collinearity, implying inversions between the two assemblies. Black dots show the detected inversion breakpoints from Fig. 1. Blue and red lines in the alignments mark the chromosomal parts used for dating the inversions, with blue indicating the ancestral and red indicating derived haplotypes.

Theory predicts that derived inversion alleles have reduced genetic variation, an elevated number of fixed differences (Hoffmann et al. 2004; Kirkpatrick 2010), and a lower number of shared polymorphisms with an outgroup (Knief et al. 2024). Following this rationale, we identified the minor inversion alleles on chromosomes *Tgu5* and *Tgu27* and the major inversion alleles on *Tgu11*, *Tgu13*, and *Tgu26* as derived. Derived homokaryotypes were identified by a significant reduction in heterozygosity (*b* = −0.003, *Z* = −9.68, *P* < 0.0001, *N* = 45 combinations of individuals homozygous for inversions from five autosomes; Fig. 2a to e) and in shared polymorphisms (*b* = −0.00005, *Z* = −3.95, *P* = 0.0003, *N* = 45; Figure S5) and a significant increase in fixed differences with a closely related species, *Poephila acuticauda* (*b* = 0.002, *Z* = 13.09, *P* < 0.0001, *N* = 45 combinations). These findings were further supported by comparisons of chromosomal collinearity between the zebra finch reference assembly and 13 closely related species with chromosome-level assemblies (see Fig. 2a to e and Table S4 and Supplementary Results). We found that the focal inversion breakpoints on chromosomes *Tgu5*, *Tgu13*, and *Tgu26* of the reference assembly were largely collinear with all other species, suggesting that these are of the ancestral type. In contrast, chromosomes *Tgu11* and *Tgu27* showed rearrangements relative to the other species (typically sharing the same breakpoints as we had previously identified), suggesting that these are of the derived type.

For chromosome *TguZ*, both homokaryotypic and heterokaryotypic individuals had a low fraction of heterozygous sites, suggesting that both alleles are derived (Fig. 2f). This observation suggests that all existing *TguZ* types are relatively recently derived and that much of the ancestral genetic diversity (visible outside of the inversion; Figure S6) has been lost. Note that *TguZ* alignments showed signs of multiple rearrangements, consistent with the presence of multiple inversions on chromosome *TguZ* (Hooper and Price 2015).

Next, we estimated divergence times for the six inversion polymorphisms (Table 1). The inversion event on *Tgu13* was the most recent, estimated to have occurred around half a million years ago, whereas the inversion events on the other four autosomes happened around 1.1 to 1.8 million years ago (Table 1). For chromosome *TguZ*, the two most common alleles were estimated to have diverged from each other around 2.2 million years ago.

### Fitness Consequences of the Inversions

For each of the six chromosomal inversions, we genotyped about 5,000 captive zebra finches using one to six SNPs in high LD with the inversion types to diagnose inversion alleles. This allowed us to quantify the fitness differences among inversion genotypes (details on tag SNPs and their selection are provided in Table S5 and Figure S7).

**Fig. 3.**
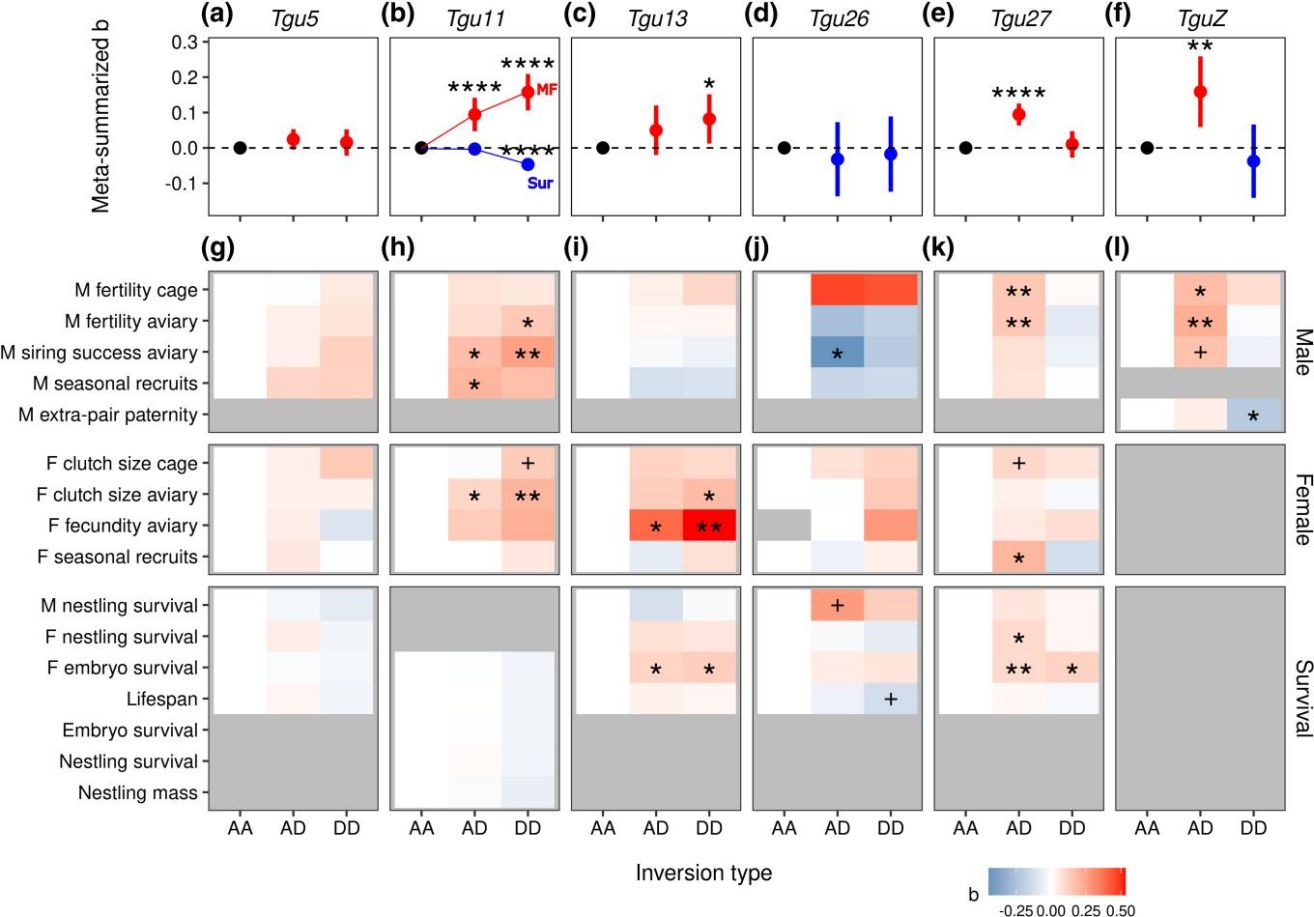
Summary of the fitness effects of the six inversions. a to f) Meta-summarized standardized effects of the inversion genotypes with their 95% CIs on fitness-related traits. Red depicts positive (beneficial) effects and blue indicates negative (detrimental) effects. Note that in b), the beneficial effects on adult reproductive performance (dots connected by a thin red line, “MF”) are depicted separately from the detrimental effects on survival (dots connected by a thin blue line, “Sur”). g to l) Estimated standardized effect sizes b of all inversion types on all tested fitness-related traits (M: male trait, F: female trait). ^+^*P* < 0.1, *P < 0.05, **P < 0.001, ***P < 0.0001; otherwise, *P* ≥ 0.1. Note that effects of *Tgu11* h) and *TguZ* l) were taken from Pei et al. (2023) and Knief et al. (2017a), respectively, where different traits were studied for that inversion type. Note that gray areas (in g to l) are traits that were not estimated for the corresponding inversion type.

As reported previously, the inversion polymorphisms on chromosomes *TguZ* and *Tgu11* exhibited weak net heterosis (Kim et al. 2017; Knief et al. 2017a; Pei et al. 2023). The inversions on *Tgu5* and *Tgu26* had no consistent, measurable effects on fitness-related traits (Fig. 3; Table S6; meta-analytically summarized standardized effects of derived genotypes compared to the ancestral homozygous genotypes ranged from −0.032 to 0.024, all *P* > 0.1). For chromosome *Tgu13*, the derived inversion showed positive additive effects on fitness (standardized mean difference between genotype AD and AA: *b*_AD_ = 0.05, *P* = 0.16; standardized mean difference between genotype DD and AA: *b*_DD_ = 0.082, *P* = 0.02), largely due to effects on female reproductive performance and survival (Fig. 3; Table S6). Chromosome *Tgu27* showed overdominance effects on most of the studied fitness-related traits. Heterokaryotypic females laid more eggs, heterokaryotypic males fertilized more eggs, and heterokaryotypic individuals had more offspring that survived until hatching and more hatchlings that survived to independence, and they lived longer in captivity (mean ± SE: *b*_AD_ = 0.094 ± 0.016, *P* < 0.0001; *b*_DD_ = 0.010 ± 0.019, *P* = 0.60; Fig. 3; Table S6).

## Discussion

### Microchromosomal Inversions

Avian species typically possess a large number of microchromosomes, which are difficult to distinguish both cytogenetically and bioinformatically. As the name suggests, these chromosomes are typically small (<2.5 µm or <20 Mb in length), acrocentric (with the centromere at one end), GC-rich, and repeat-rich. As a result, they are often lacking in genome assemblies (Fillon 1998; Pigozzi 2008) and remain poorly studied.

In our study, 9 of the 14 (putative) identified inversions are located on zebra finch microchromosomes, including *Tgu26*, *Tgu27*, and *Tgu28* (Fig. 1e and f; Figure S1c). This aligns with findings from a recent study on avian ancestral chromosome structure, which reported that avian microchromosomes *26*, *27*, and *28* had a 3-fold higher density of evolutionary breakpoint regions (i.e. breakpoints of structural variations) than the genome-wide average (Damas et al. 2018).

Overall, avian microchromosomes exhibit high gene density, show conserved gene synteny, and elevated recombination rates compared to macrochromosomes (Fillon 1998; Burt 2002; Itoh and Arnold 2005; Backström et al. 2010; Ellegren 2013; Damas et al. 2018). This study adds polymorphic inversions to the list of microchromosomal characteristics. This implies that microchromosomes may play an important role in contributing to genetic diversity, adaptation, and avian genome evolution.

### Inversion Polymorphisms Can, but Do Not Need to Be Simple

Our results suggest that inversions with a relatively simple evolutionary history and a minor allele frequency of 5% to 50% can reliably be detected using PCA on 20 wild individuals and 60 randomly selected SNPs (Figure S8). However, whole-genome sequencing (WGS) data and a complete reference assembly are better suited for detecting inversions on microchromosomes and those with lower divergence from the ancestral haplotype (Fig. 1; Figures S1 and S3). Our results further show that the ancestral and derived inversion types identified by evaluating levels of heterozygosity, number of fixed differences, and the proportion of shared SNPs with an outgroup species largely agree with those identified through multispecies alignments (Fig. 2). This suggests that in species lacking a complete reference assembly, classical population-genetic statistics can be used to infer the ancestral state of the inversion types.

The identified inversions are characterized by multiple rearrangements. With the exception of chromosome *Tgu11* (Figs. 1q and 2b; Figure S6), all contained at least one nested or overlapping inversion (Fig. 1; Figures S3 and S6). When comparing individuals homozygous for either the derived or the ancestral type, we found evidence that each inversion contained two evolutionary strata that led to opposing conclusions about ancestry (Figure S6). One stratum fitted with the overall classification of the ancestral and derived type, where the derived homozygous showed lower genetic diversity than the ancestral homozygous (estimated as the number of heterozygous sites per 50 kb sliding window) and higher levels of divergence from the long-tailed finch (estimated as more fixed differences and less shared heterozygous sites between the zebra finch and the long-tailed finch). However, a considerable part of the inversion showed the opposite signal (Figure S6). For the inversions on *Tgu5* and *Tgu13*, the switch between the two evolutionary strata coincided with the nested-inversion breakpoint detected using shared-barcode analysis, whereas on *Tgu26* and *Tgu27*, we found no such signal at the boundary between strata. Note that chromosome *TguZ* probably lost the ancestral genetic diversity within the inverted region (as shown by low genetic diversity and high divergence from the long-tailed finch; Figure S6) and contained multiple nested inversions (Fig. 2f). The inversions on *TguZ* can be considered an example of rapid evolutionary turnover potentially coupled with a higher mutation rate on *TguZ* (Meisel and Connallon 2013), which may be due to sexual selection on male-specific traits (Husby et al. 2013) in combination with a weak underdominance effect as shown by higher mortality of embryos sired by heterokaryotypic males (Knief et al. 2016). The nested inversions may further suppress gene flux between the two inversion haplotypes (when in heterozygous state). However, the overall reduction of LD toward the center of the inversions (Fig. 1k to m and p to t) suggests substantial gene flux between inversion types, either via gene conversion (Korunes and Noor 2018) or double cross-overs (Ishii and Charlesworth 1977; Stevison et al. 2011), which may restore genetic variation and help to overcome recessive deleterious load in the derived allele.

Note that the position of the inversion (and its breakpoints) does not show a clear correlation with low-recombination regions in the zebra finch as reported in Backström et al. (2010) and Singhal et al. (2015). Future studies—particularly cytogenetic observations of heterokaryotypes during meiosis—may provide further insight into how inversion influences recombination.

### Weak Heterosis as a Mechanism to Stabilize Inversion Polymorphisms

The six detected inversion polymorphisms have arisen between an estimated 0.6 to 2.2 million years ago under the assumption of neutral evolution. Although we may have missed some inversions with a low allele frequency (e.g. <5%) due to detection bias (Figure S8), all inversions have a remarkably high frequency of the derived alleles (0.395 to 0.711), both in the wild and in captivity (Table S2). Considering the distribution of the site frequency spectrum for neutral alleles, the observed frequencies are unexpectedly high. In 10^5^ permutation tests, the likelihood of randomly obtaining six allele frequencies higher than those observed was <0.023% (assuming detection at a minimal frequency of 1/2*N*, with *N* = 948 individuals) and <3.81% when limiting detection to minor allele frequencies of 40% for the derived alleles. The high frequencies of all derived alleles as well as the fact that all inversions remain polymorphic strongly suggest that the inversion polymorphisms are maintained by some form of balancing selection. Although the observed effects of the inversions on fitness-related traits were modest, they were consistent with net heterosis (mean *b*_AD_ = 0.06 and mean *b*_DD_ = 0.01; Fig. 3 and Kim et al. (2017), Knief et al. (2017a) , Pei et al. (2023)). These fitness-related traits were measured in captivity; extending analyses to wild populations would provide valuable insights into how these inversions influence individual fitness under natural conditions. Nevertheless, our results suggest that weak heterosis may be a common mechanism maintaining inversion polymorphisms and highlight that detecting this small effect requires large sample sizes.

The Australian zebra finch *T. g. castanotis* is an opportunistic breeder that is nomadic across most of the Australian continent (Zann 1996) with an estimated effective population size of one to six million individuals (Balakrishnan and Edwards 2009). The Timor subspecies *T. g. guttata* was estimated to have diverged from the Australian ones 1.9 million years ago (with 95% CI spanning from 1.2 to 2.8 MYA) likely due to a single founder event (Balakrishnan and Edwards 2009). The inversion on the sex-chromosome *TguZ* appears to be the oldest, but given the large confidence intervals around all time estimates, we cannot be confident that any inversion arose before the split of the two subspecies (Table 1). Although Itoh et al. (2011) found that *TguZ* was also polymorphic in the Timor subspecies (based on karyotypes), it remains to be seen whether the same polymorphism is shared between the subspecies. The inversion on *Tgu13* is the most recent (Table 1), and it presumably arose after the split of the two subspecies. Given the above-mentioned large effective population size of the Australian subspecies and the supposedly weak heterosis effects (Fig. 3), it seems plausible that the described polymorphisms can persist over long evolutionary timeframes at least in the Australian zebra finch (Muller 1964; Felsenstein 1974; Charlesworth et al. 1993).

Taken together, our study shows that a panmictic species with no clear ecological or population substructure contains many balanced inversion polymorphisms. Our findings highlight that both the macrochromosomes and the understudied avian microchromosomes contain polymorphic inversions that are maintained at intermediate allele frequencies. Inversion haplotype-based analyses suggest that many inversions are complex and contain nested or overlapping inversions that may further suppress gene flux between the inversion types. Dating suggests that most of the derived inversions have been segregating in the zebra finch for a long time. Although we did not detect measurable phenotypic effects for all inversions, net weak heterosis might be a common mechanism maintaining the polymorphism over extended evolutionary time.

## Materials and Methods

### Reference Genome and WGS Data

We used the most complete zebra finch assembly GCA_003957565.4 as our focal reference assembly (Rhie et al. 2021). This assembly is more complete and more contiguous compared to the previous zebra finch assembly (Warren et al. 2010); it contains four additional assembled chromosomes and the number of gaps has been reduced from 87,710 to 312 (for details, see Rhie et al. (2021)).

Details on each sample and sequencing technologies used can be found in Table S1. In brief, we used published WGS data on 27 zebra finches (26 Australian subspecies *Taeniopygia guttata castanotis* and one captive *T. g. castanotis* × *guttata* hybrid) to scan the genome for large inversion polymorphism. This included 19 wild zebra finches W1–W19 (SRA accessions ERR1013161 to ERR1013179) (Singhal et al. 2015), four captive *castanotis* zebra finches from North America (ZF1 to ZF4; i.e. 10X raw libraries for bTaeGu1–bTaeGut4 on GenomeARK) (Rhie et al. 2021), two captive *castanotis* zebra finches from captive populations in Europe, population “Seewiesen” (ZF5, ZF7), one wild-caught individual (ZF6), and one captive *castanotis* × *guttata* hybrid (ZF8; SRA accessions: SRR9671228 and SRR15012113 to SRR15012115) (Kinsella et al. 2019; Pei et al. 2022). Among these, individuals ZF1 to ZF8 were sequenced using linked-read technology and individuals W1 to W19 were sequenced using conventional Illumina WGS (Fig. 1). Note that the hybrid individual (ZF8) had 95% of its genome coming from *T. g. guttata* (Pei et al. 2022). We included this hybrid individual to maximize our sample size in the linked-read dataset. We additionally included one male long-tailed finch *P. acuticauda acuticauda* (SRA accession: ERR1013136) and one double-barred finch *Taeniopygia bichenovii* (SRA accession: ERR993524) as outgroups for phylogenetic analyses (Singhal et al. 2015).

### Raw-Read Processing, SNP Calling, and SNP Selection

For all WGS libraries, we applied a fast raw-read processing and SNP calling pipeline adapted from Pei et al. (2022), where we first mapped the raw reads against the reference using BWA-MEM v0.7.17 (Li 2013) with default settings. We mapped libraries from females and males against the reference with and without the W chromosome, respectively. Then, we called SNPs for the 19 wild zebra finches simultaneously using bcftools v1.19 mpileup (Li 2011), only taking into account reads and bases with a minimal quality score of 20. We additionally called SNPs for individuals ZF1 to ZF8 simultaneously for inversion type prediction. SNPs for the long-tailed finch and the double-barred finch were called separately for later phylogenetic analysis. We used bcftools v1.19 merge (Li 2011) to combine vcf files with the flag -0 assigning missing sites as homozygous reference.

We selected only high-quality (quality score > 100), bi-allelic SNPs using bcftools v1.19 view (Li 2011) for downstream analysis. For details, see Code and Data Accessibility.

### PCA

We coded genotypes as having 0, 1, or 2 copies of the alternative allele in comparison to the reference assembly and filtered for SNPs with a minor allele frequency above 0.1 in the 19 wild zebra finches. We performed PCA on this filtered SNP set in R V4.1.0, using the “prcomp” function from the “stats” package (R Core Team 2020). We considered any case on a given chromosome where the 19 individuals formed two to three discrete clusters along PC1 as evidence for an inversion. In case of three clusters, we expected that individuals homozygous for the two alternative inversion types would cluster at the extremes along PC1, while heterokaryotypic individuals would be intermediate. For chromosomes with only two clusters, we expected one of the clusters to contain individuals that are homozygous for the major allele and the other cluster to contain heterokaryotypic individuals. We also expected that those SNPs with the highest loading on PC1 form a single block on the focal chromosome.

We used the SNP loadings from the above PCA on the 19 wild birds to predict the PC scores of individuals ZF1 to ZF8 to infer their inversion types for downstream analyses. Note that for this step, we used only shared polymorphisms between the two zebra finch SNP sets to minimize founder effects among these mostly domesticated individuals.

### Verification of Inversion Polymorphisms Using 10X Linked-Read Sequencing Data

In order to verify and characterize the inversions, we mapped raw linked reads together with their barcodes from zebra finches ZF1 to ZF8 against the reference using the “wgs” function in Long Ranger v2.2.2 (10X Genomics). For each individual, we down-sampled the sequencing output to 35 Gb—ensuring 30X coverage—to speed up structural variant detection and to standardize library sizes.

The reference individual was homozygous for most inversions except those on *Tgu13* and *TguZ* (Figure S2), because in the new reference assembly, SNPs are phased within contigs, but not between contigs (Rhie et al. 2021). Hence, the haploid genome of the reference individual clustered in the heterozygous group on *Tgu13* and *TguZ* (Figure S2). As long as the breakpoints are assembled within contigs, this issue does not influence the identification of inversion breakpoints (Fig. 2; Figures S3 and S4). On *TguZ*, the reference was called heterozygous for types B and C (Figure S2d; note that three haplotypes of *TguZ* were identified in Knief et al. (2016)), with haplotypes B and C being closely related, derived, minor alleles). We focused on identifying the breakpoints that differentiate haplotype A (the major allele) and B plus C together (as the minor allele). We inferred the inversion types of the reference individual on *Tgu13* post hoc (i.e. based on the calls from the 10X individuals ZF1 to ZF8 in Fig. 1 and the phylogenetic analysis of the breakpoint sequence in Fig. 2).

We expected long-range barcode interactions from the two inversion breakpoints in individuals heterozygous or homozygous for the alternative types compared to the reference individual and no long-range barcode interactions in individuals that had the same inversion genotype as the reference individual. We filtered the Long Ranger output to include only intra-chromosomal barcode interactions that (i) were >0.4 Mb apart for those microchromosomes that are smaller than 20 Mb and >3 Mb apart for all other chromosomes, (ii) had a minimum quality score of four, (iii) were at least 10 kb away from an ambiguous region (i.e. multi-Ns in the reference; the default filter applied by the “BLACK_DIST” option), and (iv) were called from more than one individual to minimize false positives (but see Figure S3 for the distribution of candidates based on relaxed criteria). Long Ranger called 24 candidates from individuals ZF1 to ZF8 on chromosome *TguZ*. Because *TguZ* is known to have a large number of nested inversions (Knief et al. 2016), we only focused on the call from the most distant barcode interactions on *TguZ*.

We checked the filtered calls of intra-chromosomal barcode interactions using the 10X Genomics Loupe genome browser v2.1.1. To minimize false-positive and false-negative rates from the above filtered large structural variants that were called by Long Ranger, Yifan Pei manually scored each individual for the presence or absence of barcode interactions while being blinded for individual inversion types that had been inferred from PCA.

### Ancestral State and Divergence Time Analyses of the Inversions

We studied the evolutionary history of the six largest chromosomal inversions in the zebra finch using the filtered high-quality SNPs from the 19 wild zebra finches (W1 to W19 in Table S1). We focused on the 19 wild birds because of the homogeneity of the sample collection and sequencing methods. In contrast, the individuals ZF1 to ZF8 are of heterogeneous genetic background (most of them are domesticated birds that are closely related [e.g. ZF2 to ZF4 are a family trio] or inbred [e.g. ZF1 and ZF8] and they were sequenced using different methods). For each individual, its inversion types were inferred using PCA (Fig. 1; Figure S2). We identified regions that were inside the inversions based on (i) the distribution of SNPs with high absolute loadings on PC1 and (ii) the two most distant inversion breakpoints identified in barcode interaction analysis. SNPs with high absolute loadings on PC1 are almost always within the breakpoint regions or within the inversions identified via barcode interaction analysis. Only for the inversion breakpoint near the telomere end on *Tgu27*, SNPs with high absolute loadings on PC1 expanded 5,548 bp outside the breakpoint identified via barcode interaction analysis (Table S3). We used sequences adjacent to the first and the last SNP within the inversion as described below.

We analyzed the genetic variability within inversion genotypes (homozygous for the major allele or for the minor allele or heterozygous) to infer the ancestral and derived inversion types. We assumed that individuals homozygous for the derived inversion type would exhibit lower genetic variability and share less polymorphisms with closely related species. We first calculated for each individual the number of heterozygous sites per base pair within adjacent and equal-sized regions left and right from the breakpoints; the combined size of these region was chosen as 20% of the total length of the inversion (Figure S6). Next, we estimated the percentage of polymorphisms that were shared between each individual homozygous for one of the two inversion types and the genome of a long-tailed finch, by calculating the number of sites per base pair that are heterozygous in both the focal zebra finch individual and the long-tailed finch. The long-tailed finch has an estimated effective population size of 384,000 (Jennings and Edwards 2005) and diverged from the zebra finch ∼2.9 million years ago (Singhal et al. 2015). On each chromosome, we also included the region outside of the inversion to infer the background level of genetic variability without the inversion (see Knief et al. (2024)). Note that the right inversion breakpoint on chromosome *Tgu27* showed a low number of SNPs with high loadings on PC1, suggesting either high gene flux between the two inversion types or mis-assembly for regions near that breakpoint. Therefore, we only used the left breakpoint for the inversion on *Tgu27* (Fig. 1m).

We conducted phylogenetic analyses based on (i) the reference zebra finch assembly, (ii) wild zebra finches that were homozygous for either of the inversion types, (ii) a long-tailed finch, and (iv) a double-barred finch as the outgroup. We first created one consensus haplotype sequence using samtools v1.19 (Li et al. 2009) for each individual where heterozygous sites were re-coded following the IUPAC code. Due to the prevalence of nested and overlapping inversions (see Results), we used only one of the breakpoints for phylogenetic analysis and dating. For chromosomes *Tgu11*, *Tgu26*, *Tgu27*, and *TguZ*, we considered the called SNPs from 400 kb adjacent to the left breakpoint inside the inversion. For chromosomes *Tgu5* and *Tgu13*, we used the 400 kb adjacent to the right breakpoint to create the haplotype sequences, because the left-most breakpoint was close to the breakpoint of a second nested inversion. We built a phylogenetic tree for each chromosome using RAxML-NG v1.0.2 (Kozlov et al. 2019), assuming a general time-reversible model and a discrete GAMMA model of rate heterogeneity with 100 randomized parsimony starting trees and 1,000 bootstrap replicates. To root the tree, we used the same genomic regions from the one long-tailed finch and the one double-barred finch (LT and DB in Table S1).

The divergence times between the ancestral and derived types were estimated using beast2 v2.7.0 (Bouckaert et al. 2019). Because the linkage of SNPs decreased with increasing distance from the inversion breakpoint, we used 10 kb on autosomes and 100 kb on *TguZ* (due to its reduced genetic variation level comparing to other autosomes; Fig. 2f) from the alignments that were built for phylogenetic analysis to estimate divergence times. We used four gamma site categories and a GTR substitution model assuming a random local clock. We selected the birth-death model for MCMC priors and included one double-barred finch and one long-tailed finch as calibration points, assuming normal distributions with mean divergence times of 3.5 (SD = 0.15) and 2.9 (SD = 0.17) million years, respectively (Singhal et al. 2015). Each chromosome was run three times with 500 × 10^6^ iterations in each run. Once completed, the three runs were combined with a burn-in of 25% of the iterations to check for convergence. We considered models converging when the ESS values for the key statistics were above 200, including posterior, likelihood, prior, tree likelihood, tree height, and gamma shape. Then, the posterior estimate and the 95% highest posterior density (HPD) of the tree height between the inversion types were calculated to infer the divergence times. Note that *TguZ* had three haplotypes. We identified the haplotype based on shared SNPs described in Knief et al. (2016) and included only males that were homozygous for either A or B and females that were hemizygous for A and B.

We also used multispecies chromosomal assembly alignments to infer the ancestral state of the inversion type. For each chromosome, the zebra finch reference assembly may either represent the ancestral or the derived type. If it represents the ancestral type, we expect collinearity between the zebra finch reference and the closely related species. If it represents the derived type, we expect reversed sequence collinearity between the zebra finch reference and most of the other species, and disrupted alignments at the inversion breakpoints (identified in Fig. 1). For this analysis, we first identified the arrangement of the zebra finch reference assembly by building phylogenetic trees for each of the six inversion polymorphisms (Fig. 2) using 400 kb adjacent regions inside the inversion for one of the breakpoints (the breakpoint that was furthest away from nested/overlapping inversions, or the left breakpoint in case of a single inversion). These phylogenetic trees contained all wild zebra finches that were homozygous for either the presumably ancestral or the presumably derived inversion type (Fig. 2a to f). By incorporating the reference sequence into this tree, we identified it as homozygous (i.e. haploid) for either the presumably ancestral allele or the presumably derived allele. Next, we aligned the zebra finch reference assembly with 13 published chromosome-level songbird genomes (for details, see Table S4) using LAST v1282 (Kiełbasa et al. 2011). Multispecies alignment files were combined and visualized in R to check sequence collinearity around the identified inversion breakpoints. If the zebra finch reference haplotype was of the ancestral type, we expected to find collinearity between the zebra finch and the majority of the 13 other species. Conversely, if the zebra finch reference was of the derived inversion type, we expected to see a reverse alignment and breakage in sequence collinearity around the identified inversion breakpoints.

### Measurements of Fitness-Related Traits

We studied the effects of the inversion genotypes on the fecundity, fertility, and viability of individuals based on data from three captive populations of zebra finches maintained at the Max Planck Institute for Biological Intelligence, Seewiesen, Germany. Among them, populations (i) “Seewiesen” (population #18 in Forstmeier et al. (2007)) and (ii) “Bielefeld” (population #19 in Forstmeier et al. (2007)) were genetically independent, whereas population (iii) “Krakow” was established by cross-breeding individuals from the Krakow (#11 in Forstmeier et al. (2007)) and from the “Seewiesen” populations. For details, see Pei (裴一凡) et al. (2020).

Data on reproductive performance and lifespan were taken from https://osf.io/tgsz8/ (Pei (裴一凡) et al. 2020). Overall, reproductive performance of birds was measured either in cages with a single breeding pair, or in aviaries where a group of males and females could form breeding pairs freely. For detailed information on experiment setups, rearing conditions and measurements of traits, see Pei (裴一凡) et al. (2020). In brief, for female fecundity, we measured the number of eggs per clutch in cages and in aviaries. For male fertility, we measured (i) whether an egg laid in a cage or in an aviary was fertilized by the social male or not and (ii) the total number of eggs a male fertilized, including both within- and extra-pair eggs. For each male and female adult, we also measured offspring survival, as (i) whether a fertilized egg (with an embryo) hatched or not and (ii) whether a chick reared by the focal parent survived until independence (i.e. 35 days of age) or not. For male and female fitness, we measured the total number of independent young produced by the focal (genetic) mother and the total number of independent young sired by the focal father. Lifespan of an individual was measured as the number of days from the date of hatching until death.

### Tag SNP Selection, Genotyping, and Allele Frequency Calculation

To characterize the inversion genotype for a large number of individuals, we used the tag SNPs as a proxy. High-quality tag SNPs were selected before the generation of sequencing data (see also *Whole-genome sequencing data*). For details on SNP positions see Table S5 and on tag SNP selection see Materials and Methods. In brief, one to six SNPs per chromosome that had low missing rates and that were in high LD with the inversion type among 948 wild zebra finches (Knief et al. 2016) were selected to tag the inversion types for chromosomes *Tgu5*, *Tgu11*, *Tgu13*, and *TguZ*. For the inversions on the two microchromosomes *Tgu26* and *Tgu27*, two candidate SNPs were first selected based on their high loadings on PC1 in the 948 wild zebra finches (i.e. based on the information available at that time). Then, we selected the SNP in highest LD among the 19 wild zebra finches with the inversion types on *Tgu26* and *Tgu27* to tag the inversions for our fitness analysis (Figures S7a and g). Simulations show that tag SNPs that are in lower LD with the inversion type would lead to an underestimation of the true heterosis effect or to the conclusion that the effect would not be significant (Figure S9). Hence, potential misclassifications would not lead to spurious results, and the estimated heterosis effects we report here are—if anything—an underestimate of the true heterosis effects.

For 4,958 zebra finches from the three captive populations (see above), a total of 15 tag SNPs (three on *Tgu5* and *Tgu13*; one on *Tgu11*, *Tgu26*, and *Tgu27*; and six on *TguZ*) were genotyped using the Sequenom MassARRAY iPLEX platform (Knief et al. 2015) at the Institute of Clinical Molecular Biology at Kiel University, Germany. Genotypes were called by the default parameters on the MassARRAY iPLEX platform (see Knief et al. (2017b) for details). To study the effect of the *Tgu11* inversion genotype on offspring survival (Pei et al. 2023), an additional 1955 birds, 3022 dead embryos, and 1522 dead nestlings were genotyped for one tag SNP using the Roche LightCycler Instrument following the manufacturer's guide. Inversion calls for *Tgu5*, *Tgu11*, *Tgu13*, and *TguZ* were taken from Knief et al. (2015, 2016, 2017a) and Pei et al. (2023).

The allele frequencies of inversions among the 19 wild zebra finches with WGS data were estimated based on haplotype frequencies. For captive birds, allele frequencies were estimated using the frequencies of the tag SNPs designed above. Because the Australian zebra finch is a panmictic species with no known population substructure, these estimated frequencies should represent the species as a whole.

### Statistical Analyses

We used mixed-effect models to estimate the effects of inversion genotypes on all fitness-related traits. All models were fitted using the function “lmer” in the R package “lme4” (Bates et al. 2015). Links to detailed model structures, all scripts and data can be found in the Code and Data Accessibility statement (also see Table S6). To compare and summarize the effect sizes of the genotypes on the different fitness-related traits, we treated all response variables as normally distributed and Z-scaled them. We estimated the genotypic effects of each of the inversion types in separate models. Specifically, for each chromosome, instead of fitting individual genotypes as a factor with multiple levels, we treated the homozygous ancestral allele “AA” as baseline (Fig. 3). Thus, to estimate the effect of the inversion genotype, we compared each fitness-related trait between the ancestral homozygotes and either the heterozygotes (“AD”) or the homozygotes for the derived allele (“DD”) (Fig. 3). We controlled for the effects of inbreeding (using the pedigree-based inbreeding coefficient) and age (in days) by fitting them as additional covariates whenever possible. Additionally, we controlled for the confounding fixed effects (i) laying order, (ii) hatching order, and (iii) clutch order within the breeding season, (iv) whether the clutch was laid while nestlings from a previous clutch were still in the nest (yes/no), and (v) population (three levels: Seewiesen, Bielefeld, Krakow), by including them as factors whenever applicable. To control for pseudo-replication we included female, male, pair, clutch, and breeding season identity as random effects, whenever applicable.

To meta-summarize the genotype effect on all fitness-related traits, we used weighted linear models for each chromosome separately using the “lm” function in the R package “stats” (R Core Team 2020). Using the above estimated standardized effect sizes of the genotypes as response variables, we fitted the genotype as a fixed factor with two levels (because the genotype of the ancestral homozygous AA was the baseline and was not estimated in the mixed-effect models). To account for the uncertainty of the estimated effect sizes, we included the multiplicative inverse of the standard error of each estimate as the weight. We removed the intercept to estimate the mean effect sizes for the heterozygous AD and homozygous DD genotypes.

## Supplementary Material

msaf262_Supplementary_Data

## Data Availability

WGS data for zebra finches were obtained from the NCBI SRA under accessions ERR1013161 to ERR1013179 (Singhal et al. 2015), SRR9671228 (Kinsella et al. 2019), and SRR15012113 to SRR15012115 (Pei et al. 2022), as well as four additional datasets (bTaeGut1 to bTaeGut4) available via GenomeARK (Rhie et al. 2021). WGS data for the outgroup species include one male long-tailed finch (*P. acuticauda acuticauda*; ERR1013136) and one double-barred finch (*T. bichenovii*; ERR993524; Singhal et al. 2015). Details of all WGS datasets are provided in Table S1, and chromosomal assemblies used in this study are listed in Table S4.

## Code and Data Accessibility

All supporting data including SNP genotype data and scripts can be found at https://osf.io/m2yrf/ at the Open Science Framework. Fitness-related data can be found at https://osf.io/tgsz8/.
