# Supplementary material for: Evolution of Large Polymorphic Inversions in a Panmictic Songbird
Source: Mol Biol Evol. 2025 Oct 16;42(11):msaf262. doi: 10.1093/molbev/msaf262 (PMC12612816; doi:10.1093/molbev/msaf262)
Supplement: msaf262_Supplementary_Data [file msaf262_supplementary_data.zip › MBE-25-0243_allinvs_SupplementaryTextFigs_correctedFigS5_Final.pdf]

**Supplementary text and figures for**

# Evolution of large polymorphic inversions in a panmictic songbird

**Pei *et al.***

## Supplementary Text

### Supplementary methods

*10X reads barcode interaction analysis*

Note that we used bTaeGut1.pri.cur.20210409 as our reference to identify inversion breakpoints. Note that the focal assembly GCA_003957565 is a slightly polished version of the earlier version i.e. bTaeGut1.pri.cur.20210409 with all major chromosomes nearly identical (**Supplementary Table S7**). We anyway later used BLAT v36x2 to lift the breakpoint regions detected via barcode interaction to our focal assembly and they were identical.

To check for false-positive signals (e.g. due to mis-assembly or repetitive elements in the reference) and false-negative calls (e.g. coverage of barcodes below detection threshold), we manually called from **Supplementary Fig. S3** the absence (i.e. being identical to the reference haplotype) and presence (i.e. heterozygous or homozygous for the alternative haplotype) of barcode interactions in all eight individuals, for each of the large intra-chromosomal interactions called by Long Ranger. For all chromosomes but *TguZ*, the resulting calls (**Figs 1P-T**, shown in red) indeed matched the inversion types of each individual that were predicted from PCA (**Figs 1P-T, S2** and **Figs S3A-F**). For the distant barcode-interactions on chromosome *TguZ*, the barcode interactions were found in all individuals regardless of their inversion type (**Fig 1U,** and **Figs S3G, H**). Moreover, on *TguZ*, the barcode interaction patterns do not resemble those from other chromosomes (**Fig. S3**). This suggests that the true breakpoints on *TguZ* might contain repetitive sequences and that they are still missing from the reference.

*Tag-SNP selection*

For the selection of tag SNPs on *Tgu26* and *Tgu27*, we had the following information available. Knief *et al.* (2016) (Knief et al. 2016) genotyped 948 wild Australian zebra finches, 88 founders of the Seewiesen population, 74 founders of the Bielefeld population and 63 founders of the Krakow population using an Illumina Infinium iSelect HD Custom BeadChip. Among the 4553 genotyped SNPs, 34 and 36 were evenly spaced along *Tgu26* and *Tgu27*, respectively (**Supplementary Figs S7A, G**). Knief *et al.* (2016) (Knief et al. 2016) detected weak signals of

linkage disequilibrium on *Tgu26* and *Tgu27* among the 948 wild zebra finches through principal component analysis with maximal $r^2$ values of 0.244 and 0.553 (**Fig. S2**), respectively. They concluded that there might have been polymorphic inversions on these two chromosomes that were either not tagged perfectly by the SNPs or that had exchanged material between inversion types through gene conversion (Korunes and Noor 2018) or double crossovers (Ishii and Charlesworth 1977; Stevison et al. 2011). For each of these two microchromosomes, two tag SNPs were selected based on their loadings on PC1 in the 948 wild zebra finches (i.e. based on the information available at that time). For details on SNP positions see **Supplementary Table S5**. The correlations (i.e. linkage disequilibrium) between the genotypes of these tag SNPs with the (later-identified) inversion types among the 19 wild birds were high on *Tgu27* (Pearson's correlation coefficients $r^2 = 0.83$ and 0.81, respectively; $P < 0.0001$; **Supplementary Table S4** and **Figs S7G–L**). On *Tgu26*, one tag SNP was in high LD (Pearson's correlation coefficients $r^2 = 1.00$; $P <= 0.001$) whereas the second SNP was not in LD with the inversion type (Pearson's correlation coefficients $r^2 = 0.30$; $P >= 0.2$; **Supplementary Table S4** and **Figs S7A–F**). Next, we selected the SNP in highest LD among the 19 wild zebra finches with the inversion types on *Tgu26* and *Tgu27* for our fitness analysis.

## Supplementary results

*Ancestral state analysis*

Assuming the simplest scenario where an inversion polymorphism goes back to a single mutational event, we expect the derived inversion haplotype to be largely depleted of genetic variation and to contain more fixed differences in comparison to an outgroup (Hoffmann et al. 2004; Kirkpatrick 2010). This is because all copies go back to the same founder and because recombination with the genetically diverse ancestral haplotype is largely suppressed (Hoffmann et al. 2004; Kirkpatrick 2010). Thus, the ancestral arrangement should have more heterozygous sites and share more ancestral polymorphism with a closely related species (Knief et al. 2024). Moreover, this pattern should be most pronounced in regions close to the breakpoints, where recombination between the two arrangements is suppressed the most (see **Figs 1H–U**).

Following our above predictions on genetic diversity, we concluded that the minor inversion alleles on chromosomes *Tgu5* and *Tgu27* and the major inversion alleles on *Tgu11*, *Tgu13*, *Tgu26* are derived (**Table 1**). Specifically, individuals homozygous for the minor inversion alleles on chromosomes *Tgu5* and *Tgu27* and those homozygous for the major alleles on the other three autosomes had (1) a significantly lower fraction of heterozygous sites compared to those homozygous for the other allele (b = -0.003, Z = -9.68, P < 0.0001, N = 45 combinations of individuals homozygous for inversions from five autosomes), (2) a significantly lower fraction of shared polymorphisms (b = -0.009, Z = -4.49, P < 0.0001, N = 45 combinations) and a significantly higher amount of fixed differences with a long-tailed finch *Poephila acuticauda acuticauda* in comparison to the ancestral allele (b = 0.002, Z = 13.09, P < 0.0001, N = 45 combinations, **Figs 2, S5**). For each individual, the above three measures were highly correlated (|r| > 0.53, |Z| > 8.77, P < 0.0001, N = 45, controlling for the inversion type as a fixed effect, and chromosome ID and individual library ID as random effects). For chromosome *TguZ*, both homokaryotypic and heterokaryotypic individuals had a low fraction of heterozygous sites, implying that both alleles are derived (**Fig. 2F**). This observation suggests that all existing *TguZ* types are relatively recently derived and that much of the ancestral genetic diversity (visible outside of the inversion, **Fig. S6**) has been lost.

Overall, we found that individuals heterokaryotypic for inversions had higher fractions of heterozygous sites in the inverted regions than homokaryotypic individuals (b = 0.005, Z = 16.80, P < 0.0001, N = 104 combinations of 19 individuals and 6 inversions; **Fig. 2**). This is expected due to the reduced recombination and the accumulated fixed-differences between the two inversion types (Kirkpatrick 2010).

As an alternative approach to identify the ancestral and derived types, we compared the chromosomal collinearity between the zebra finch reference assembly and 13 closely related songbird species with chromosome-level assemblies (for details of assemblies see

**Supplementary Table S4**). When comparing the pairwise alignments between the zebra finch reference assembly and closely related species, we found that the focal inversion breakpoints on chromosomes *Tgu5, Tgu13* and *Tgu26* of the reference assembly were largely collinear with all other species, suggesting that these are of the ancestral type. In contrast, chromosomes *Tgu11* and *Tgu27* showed rearrangements relative to the other species (typically sharing the same breakpoints as we had previously identified), suggesting that these are of the derived inversion type.

This approach confirmed our population genetic identification of the derived inversion type for the five autosomes (**Fig. 2**). For chromosome *TguZ,* alignments showed signs of multiple rearrangements, consistent with the presence of multiple inversions on chromosome *TguZ* (Hooper and Price 2015). The multispecies alignments of chromosomes *Tgu26, Tgu27* and *TguZ* were generally less consistent than those of the other three chromosomes, suggesting either that these chromosomes are especially prone to structural rearrangements (e.g. due to the higher recombination rate on the microchromosomes (Backström et al. 2010)), or that the assemblies for these chromosomes were not as complete as for the other ones.

We estimated the divergence time between the two arrangements for each of the six inversion polymorphisms (**Table 1**), analyzing 10 kb for the five autosomes and 100 kb for *TguZ* (due to the limited genetic variation within each *TguZ* haplotype). These regions were located adjacent to the same breakpoints used to construct the phylogenetic trees. The inversion event on *Tgu13* was the most recent, estimated to date back to about half a million years ago, whereas the inversion events on the other five autosomes happened around 1.1-2.2 million years ago (**Table 1**). For chromosome *TguZ*, the two most common alleles were estimated to have diverged around 2.2 million years ago.

## Supplementary figures

**Fig. S1.** PCA plots for the 19 wild zebra finches and absolute loadings on PC1 for additional chromosomes that show two or three clusters of individuals along PC1. Among them, eight chromosomes *Tgu7, Tgu8, Tgu28* to *Tgu31*, *Tgu33* and *Tgu34* (**A-F, H** and **I)** show signs of a putative inversion polymorphism with two haplotypes (i.e. birds are grouped into two to three clusters along PC1 and the distribution of SNPs with high absolute loading on PC1 are clustered in large blocks). However, for the remaining two chromosomes *Tgu32* and *Tgu37*, their evidences to contain putative inversions are less convincing, because the distribution of SNPs with high loadings do not show gradual changes along the physical position on the chromosome. For additional details see legend of **Fig. 1.** And for PCA plots for all other autosomes that do not show any pattern of putative structural variations please see **Figs S10**, **S11**.

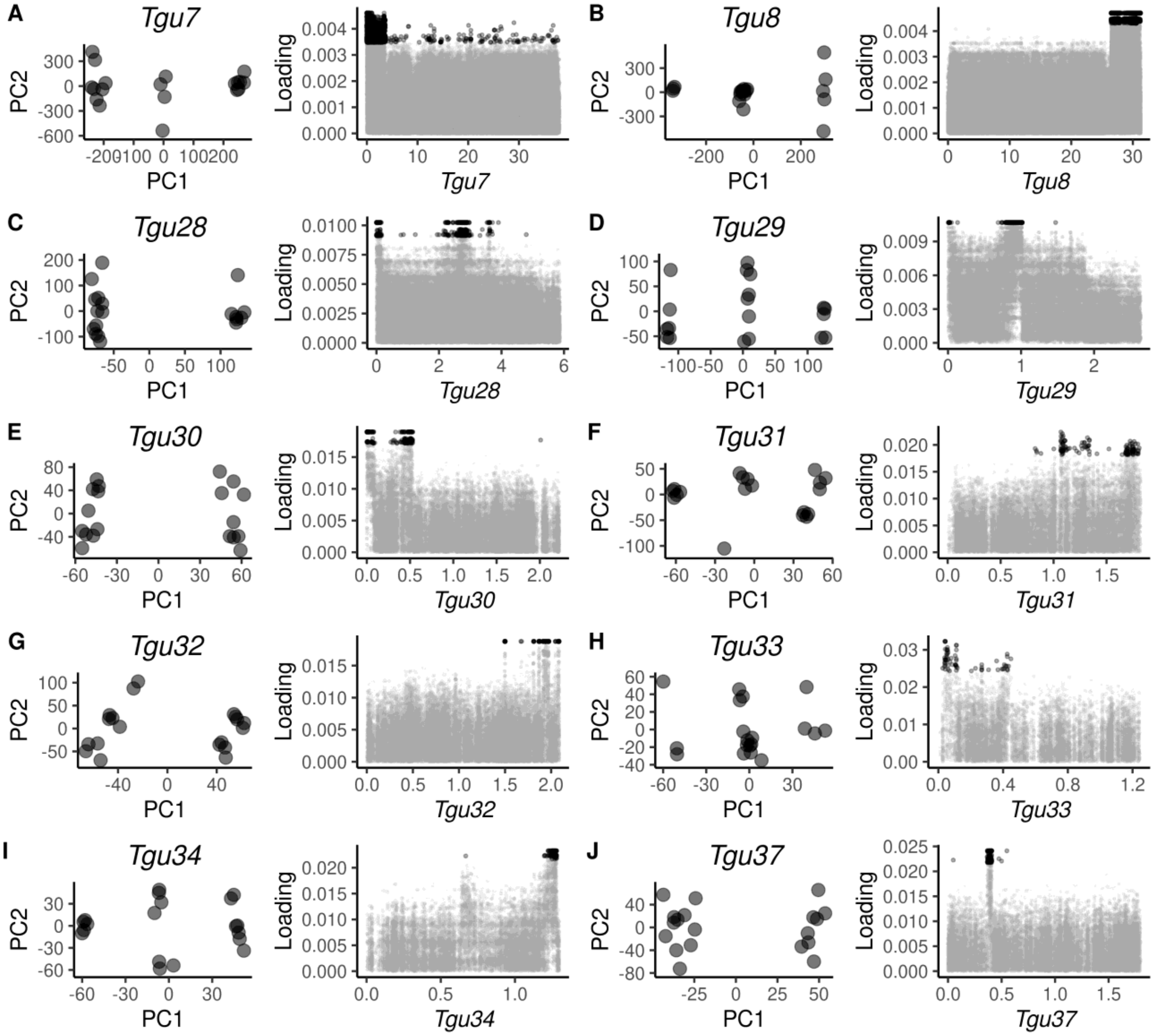

**Fig. S2.** Principal component analysis based on the 4553 randomly selected SNPs (genome-wide) genotypes from 948 wild zebra finches (small grey dots) used in (Knief et al. 2016) for chromosomes that contain inversion polymorphisms, i.e. *Tgu5*, *Tgu11*, *Tgu13*, *TguZ*, *Tgu26* and *Tgu27*. Dots represent wild-caught individuals whereas triangles are captive individuals (**Methods**). The PC values of the 27 zebra finches used in this study (red, blue and black) and the zebra reference assembly used in this study (Rhie et al. 2021) (orange) were predicted by the loadings of the 4553 SNPs from the 948 wild birds. Red and blue depict individuals that are homozygous for the minor or major alleles, respectively (**Fig. 1**), whereas black indicates heterozygous individuals. Grey letters A, B and C indicate the inversion types of the major, minor and the least abundant alleles defined in (Knief et al. 2016). Individuals used for linked-read analysis are highlighted by boxes around their IDs (**Figs 1, S2**). For a detailed description of haplotypes defined in this study and (Knief et al. 2016) see **Table 1** and **Supplementary Table S2**. For additional information of the methods also see (Pei 2022), PhD thesis. Note that, due to the low number of SNPs on the microchromosomes, (Knief et al. 2016) we lacked the power to detect inversion signals on *Tgu26* (**E**) and *Tgu27* (**F**) - hence the absence of clear clustering along PC1 in these two panels.

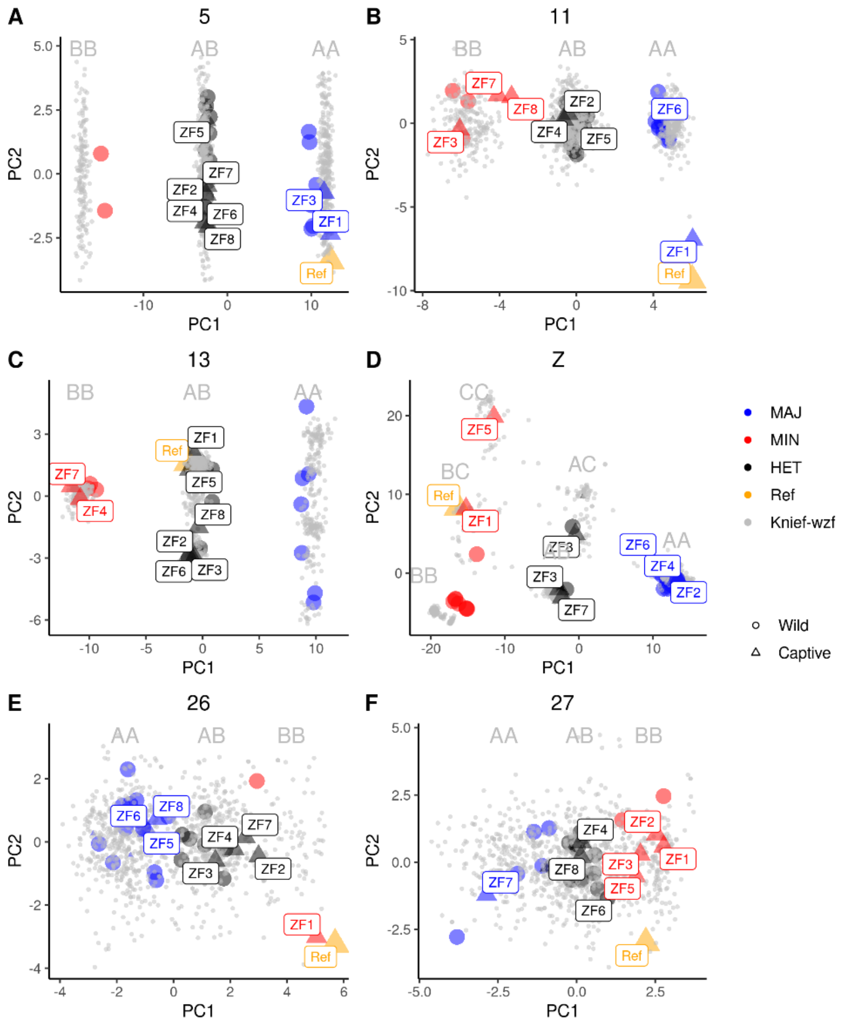

**Fig. S3.** Genome-wide distribution of unexpected intra-chromosomal barcode interactions that were detected by Long Ranger (blue and light blue curves). All individuals (rows) are *Taeniopygia guttata castanotis*, except for individual ZF8 which is a hybrid between the two subspecies *T. g. castanotis* and *T. g. guttata* (see **Methods**; also see (Knief et al. 2015; Pei et al. 2022)). Chromosomal positions are based on the new genome assembly bTaeGut1.202104 (Rhie et al. 2021). Blue depicts all high-quality candidate calls of intra-chromosomal barcode interactions that are more than 0.4 Mb apart for microchromosomes shorter than 20 Mb and more than 3 Mb for the others (i.e. identical to the yellow ones in **Fig. 1**; also see **Fig. 1** and Methods in the main text). Sky blue depicts candidate calls with relaxed threshold (i.e. next to gaps in the assembly). Blue numbers indicate the expected number of alternative inversion types based on PCA of SNPs (**Supplementary Fig. S2**). Each vertical bar is a SNP that was used in (Knief et al. 2016). SNP positions were lifted from the old TaeGut1 (Warren et al. 2010) to the new assembly (Rhie et al. 2021). The blackness of each bar indicates the highest level of linkage disequilibrium (LD) of this SNP with another SNP that is at least 1 Mb apart within the same chromosome, among 948 wild zebra finches (Knief et al. 2016). The large blocks of SNPs on chromosomes *Tgu5*, *11*, *13*, *Z*, that are in high LD (black bars) indicate the inversions, whereas the microchromosomes *Tgu26* and *Tgu27* contained weak signals of LD, which was suggestive of inversion (Knief et al. 2016). Note that the distant barcode interactions detected by Long Ranger were enriched for chromosomes that contained inversions (i.e. N=40 blue curves on chromosomes that show a signal of segregating inversions, i.e. *Tgu5*, *11*, *13*, *26*, *27* and *Z*; N = 19 blue curves on chromosomes *Tgu7*, *Tgu28*, *Tgu28*, *Tgu30* and *Tgu34* that with a putative inversion; N =9 blue curves on chromosomes *Tgu32* and *37* that show somewhat signals with putative inversions; and lastly N = 5 were found on the remaining chromosomes *Tgu2, 16, 19* and *25*). The missing calls in individuals with one or two copies of the derived allele are due to false negatives.

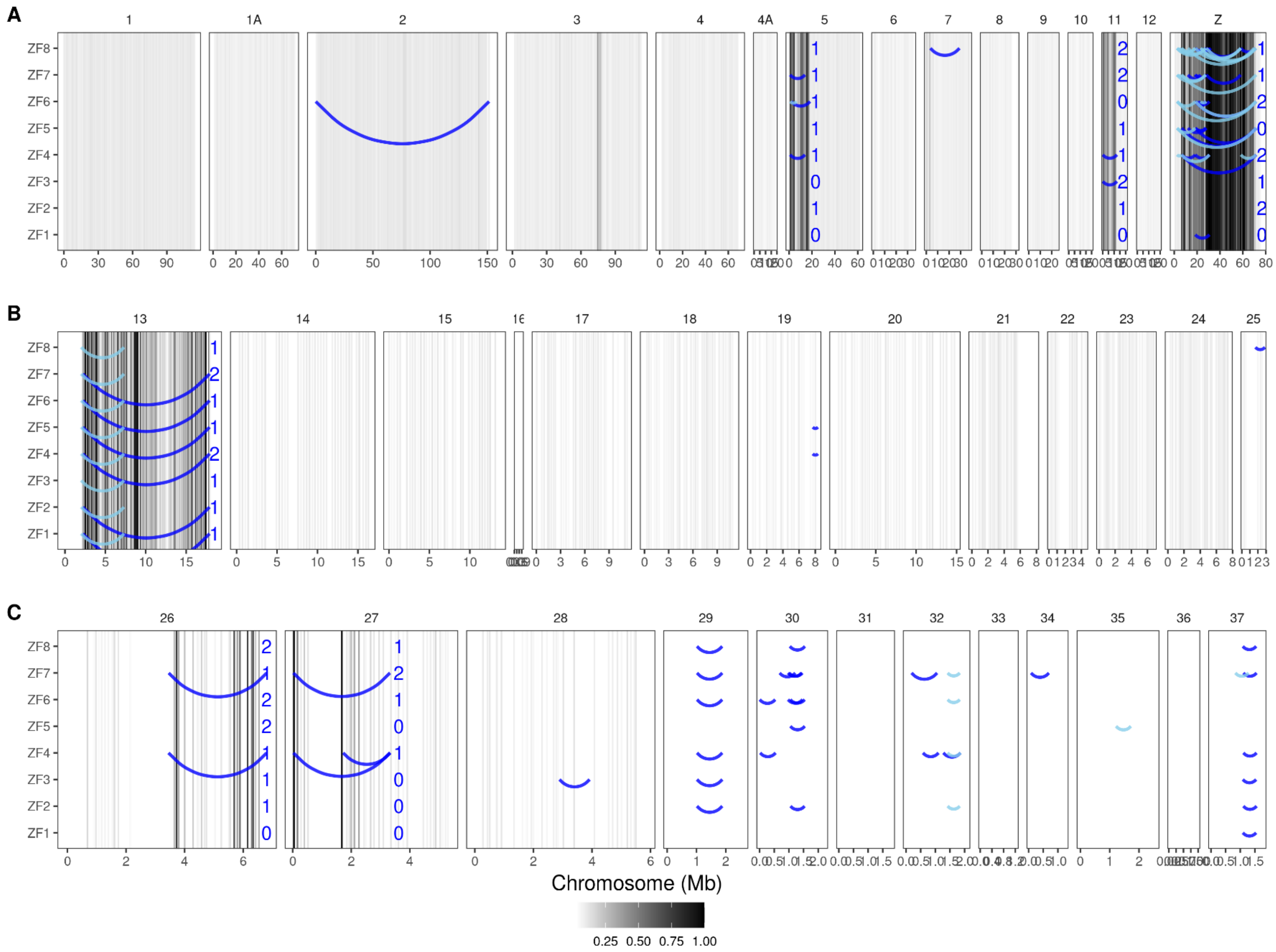


163

**Fig. S4.** Heat map of the number of $log_2$-transformed barcodes shared between distinct intra-chromosomal regions (i.e. putative inversion breakpoints) detected by Long Ranger (**Supplementary Table S3;** for details see **Methods**). Dots represent overlapping barcodes. The increasing number of overlapping barcodes towards the two distinct intra-chromosomal regions suggests that the sample contains at least one alternative inversion compared to the reference. The point with the highest number of overlapping barcodes indicates the breakpoints. Samples that contain a signal for at least one alternative inversion type were marked by red frames. Genomic positions were based on the reference assembly GCA_003957565 (Rhie et al. 2021)**.**

173

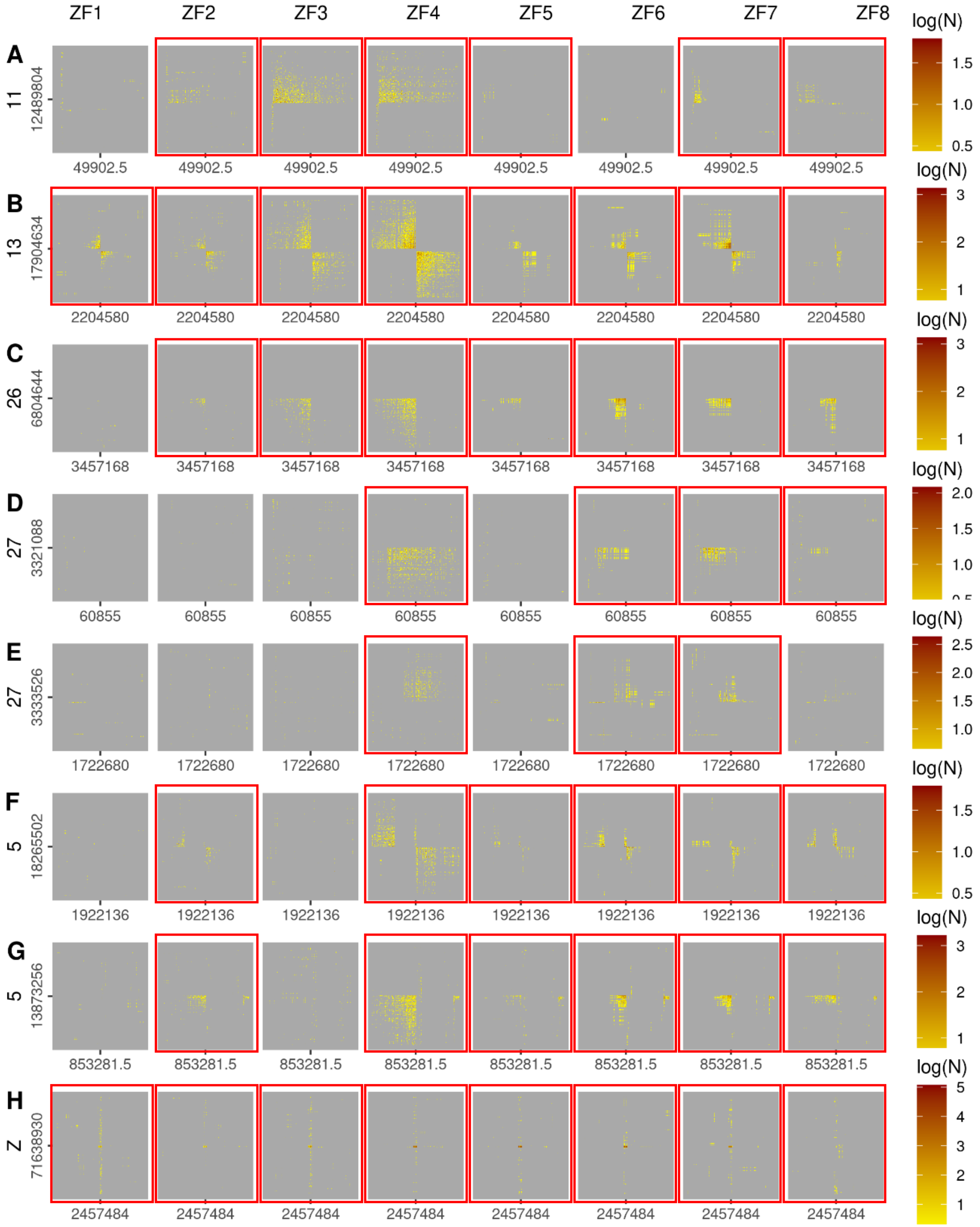
ZF1
ZF2
ZF3
ZF4
ZF5
ZF6
ZF7
ZF8
A
11
12489804
49902.5
B
13
17904634
2204580
C
26
6804644
3457168
D
27
3321088
60855
E
27
3333526
1722680
F
5
18265502
1922136
G
5
13873256
853281.5
H
Z
71638930
2457484
log(N)

**Fig S5.** Histogram of the number of shared heterozygous sites per base pair between each of the 19 wild zebra finches and the long-tailed finch. For additional details see **Fig. 2.** Het-BC (**F**) indicates the individual that is heterokarotypic for inversion types B and C for *TguZ.*

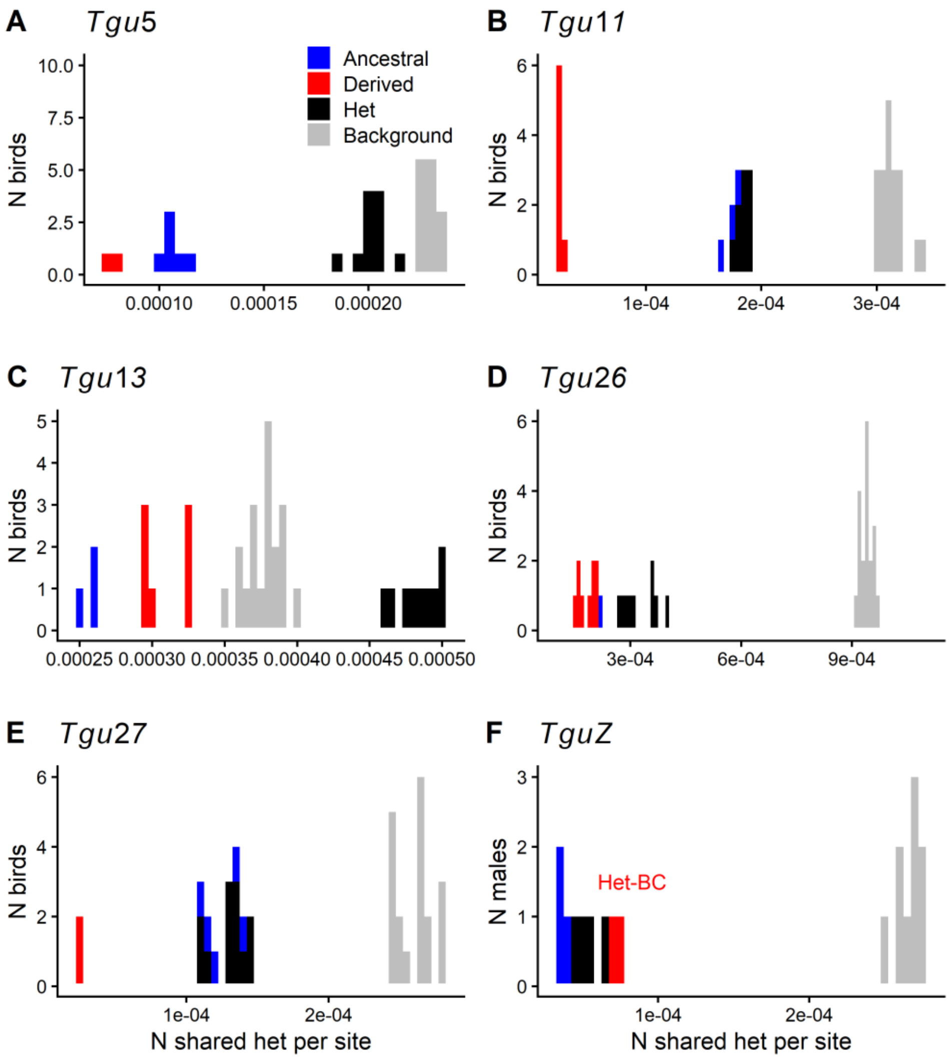

**Fig. S6.** Individual based population genetic analysis for individuals (W1 to W19) that are homozygous for either of the inversions on chromosomes *Tgu5*, *Tgu11*, *TguZ, Tgu13*, *Tgu26* and *Tgu27* (left to right). For each chromosome, top to bottom panels are the number of fixed differences, the number of shared heterozygous sites between the zebra finch and a long-tailed finch, the total number of heterozygous sites as well as the fraction of shared heterozygous sites with a long-tailed finch. These values were estimated in 50 kb non overlapping sliding windows (red and blue dots, smoothed by individual per line). Red indicates individuals that are homozygous for the more derived inversion type whereas blue shows homozygous for the ancestral type (or less derived inversion type for *TguZ*). Note that the overall ancestral or derived state for each chromosome was estimated based on the regions right next to the two most distinct breakpoints (with orange shading; details see **Materials and Methods**). Black lines are inversion breakpoints identified in **Fig. 1**. Note that *Tgu11* had the simplest evolutionary history of one inversion whereas the others all seemed to have at least one shifting of the ancestral versus derived state, suggesting potential nested and overlapping inversions. Intriguingly, *Tgu13*, *Tgu26* and *Tgu27* all showed two contradicting signals at the two ends of the inversion.

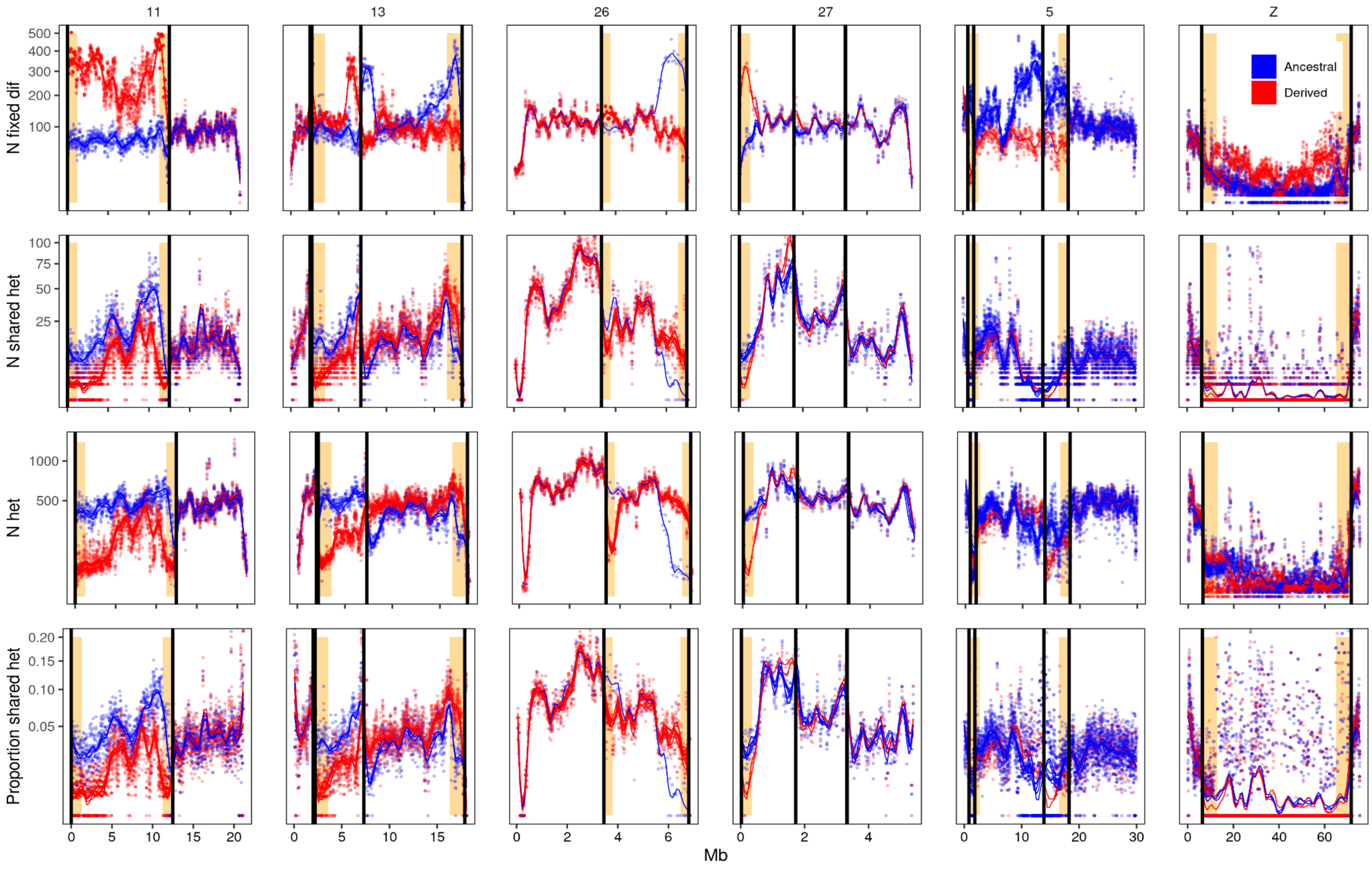

11
13
26
27
5
Z
Ancestral
Derived
N fixed dif
N shared het
N het
Proportion shared het
Mb

**Fig. S7.** Tag SNPs (red dots in **A,D,G,J**) for the inversions on microchromosomes *Tgu26* (**A-F**) and *Tgu27* (**G-L**) were selected based on the highest loadings on PC1 from PCA analysis of 34 (**A-C**) and 36 SNPs (**G-I**) among the 948 wild zebra finches used in (Knief et al. 2016) (**A-C** and **G-I**). PCA analyses using SNPs from WGS data from the 19 wild zebra finches (Singhal et al. 2015) (**D,J**) show that the selected tag SNPs (red dots in **A,D,G,J**) were in high LD with the defined inversion types (**E,K,L**) except for the tag SNP on *Tgu26* at 2.5 Mb that was in relatively low LD (**F**; for additional details see also **Supplementary Table S5**). Red boxes highlight the positions of the tag SNPs (**A,D,G,J**). (**B,C,E,F,H,I,K,L**) In PCA plots, blue depicts the tag SNP genotype of individuals that are homozygous for the major allele homozygous (MAJ), red depicts individuals homozygous for the minor allele (MIN) and black indicates heterozygous individuals (HET). A and B are based on the major and minor alleles among the 948 wild zebra finches in (Knief et al. 2016). Note that the SNP at 3,097,302 bp on reference taeGut1 (Warren et al. 2010) on *Tgu27* showed heterosis on all fitness-related traits (**Fig. 3**). For additional information of the methods also see (Pei 2022), PhD thesis .

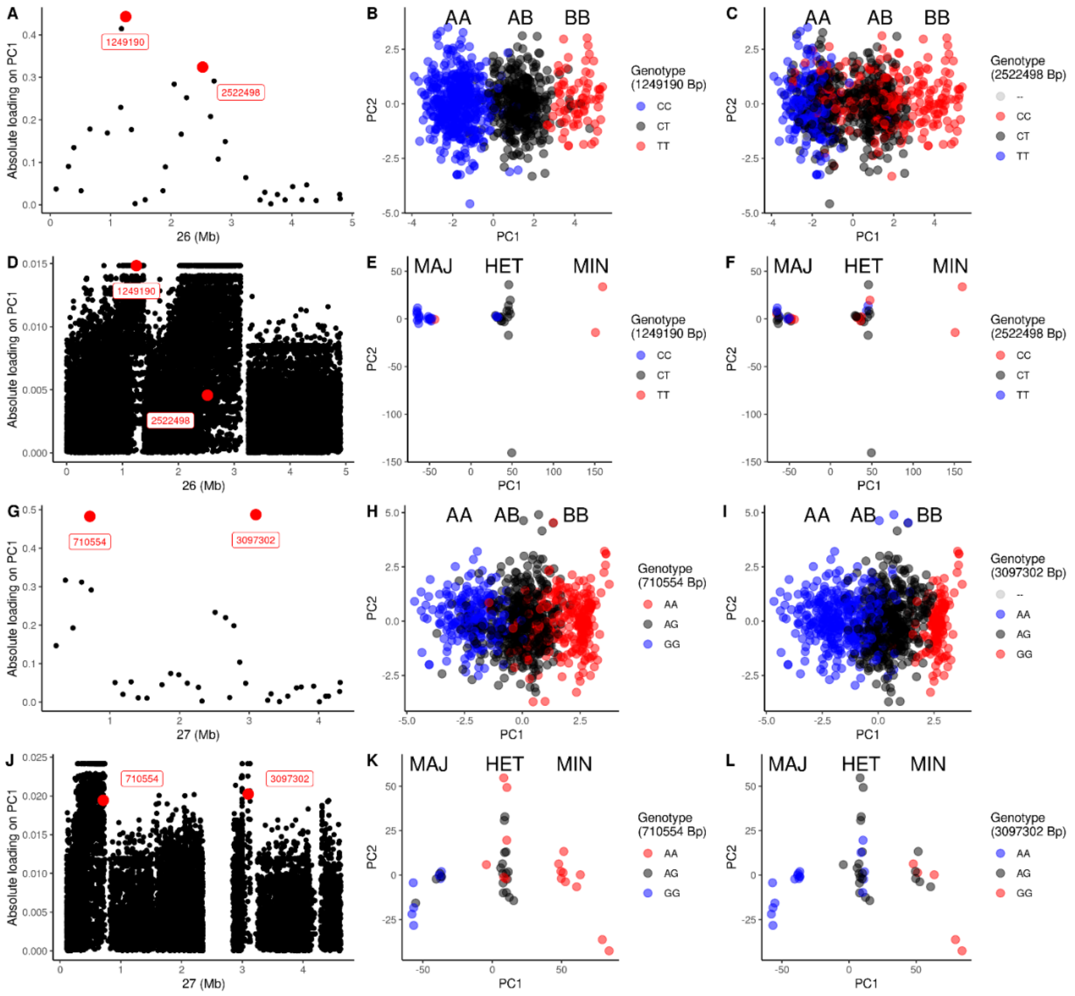

**Fig. S8** Inversion detection efficiency using PCA when allele frequency of the inversion ranged from 0.01 to 0.99 (x panels) and number of individual genotyped ranged from 10 to 1000 (y panels). SNP data of 948 wild zebra finches on *Tgu11* that contain a single inversion polymorphism with intermediate allele frequency were taken from (Knief et al. 2016). For each combination of the number of birds and allele frequency of the inversion, SNPs were randomly selected, and a PCA was computed. Note that overall inversion polymorphism can quite reliably detect with minor allele frequency > 0.01 if the sample size is large enough (e.g. > 600 birds).

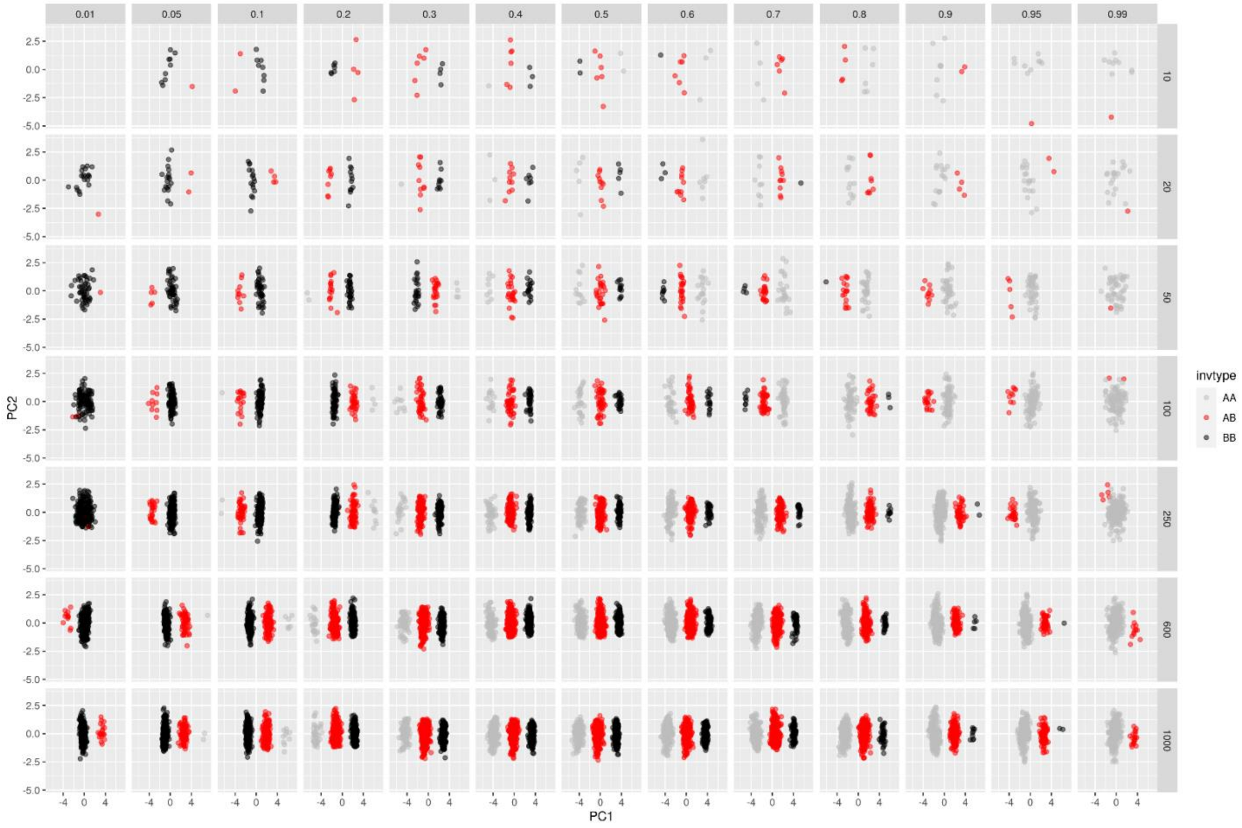

**Fig. S9.** Estimated effect sizes decrease when linkage disequilibrium (LD) between the tag SNP and the derived inversion type decreases due to (**A**) random assignment of genotypes (e.g., recombination or gene conversion between the inversion types), (**B**) the tag SNP present in a subset of the derived haplotype, and (**C**) failure to call a true heterozygous genotype (e.g. due to genotyping error). To examine the LD between tag SNP and the estimated effect size, we here simulated a population of 10,000 individuals with a polymorphic inversion under Hardy–Weinberg disequilibrium that shows weak heterozygous advantage (i.e., heterokaryotypic individuals AD show 0.1 higher fitness). The genotype frequencies of the inversion karyotypes are 0.25, 0.5, and 0.25 for homokaryotypic ancestral AA, heterokaryotypics AD, and homokaryotypic derived types DD, respectively. We then simulated the tag SNP genotypes to generate different levels of LD with the true inversion genotype, where LD = 1 indicates complete linkage between the tag SNP and the inversion. Each scenario was simulated 100 times.Note that reduced LD results in either a smaller observed effect size compared to the true effect size of 0.1 (blue horizontal dashed line) and/or insignificant findings (indicated by overlap of the 95% CI with zero, shown by the black dashed line). For our tag SNP on *Tgu27*, the estimated LD with the inversion type is 0.83 (red vertical dashed line), where the estimated effect size is smaller than the expected true effect size in our simulations. The corresponding R script is available in the Supporting Data and Scripts.

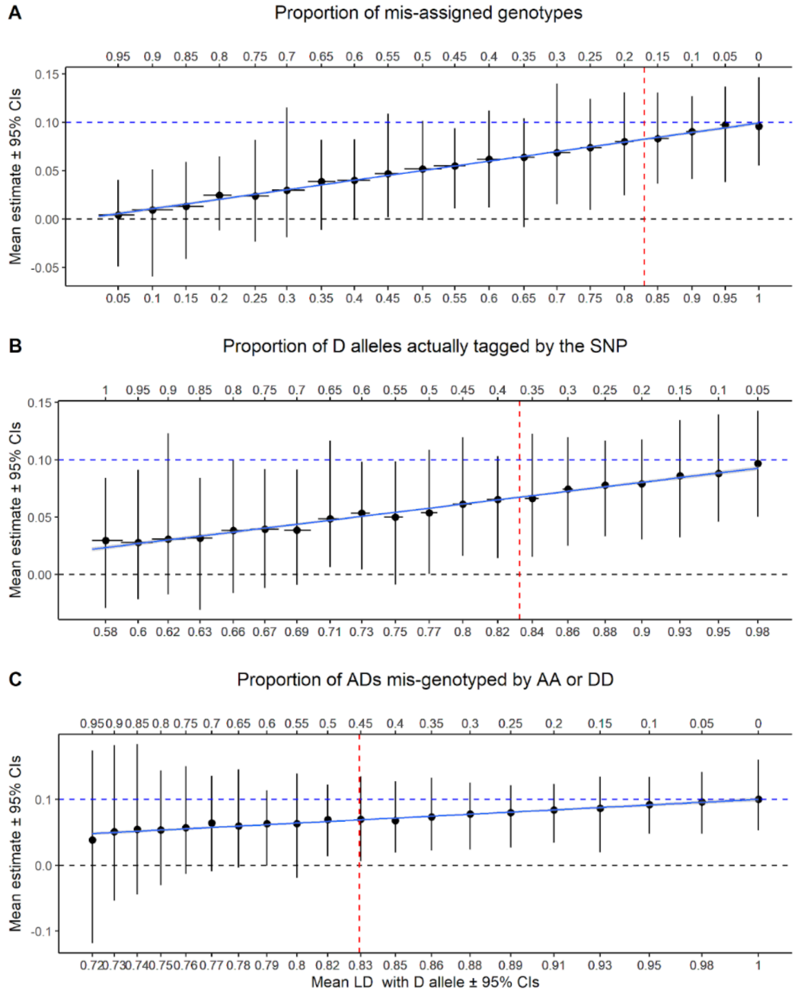

**Fig. S10.** Absolute loadings on PC1 for chromosomes (in Mb) that show no signal of chromosomal inversions**.** For additional details see legend of **Fig. 1**.

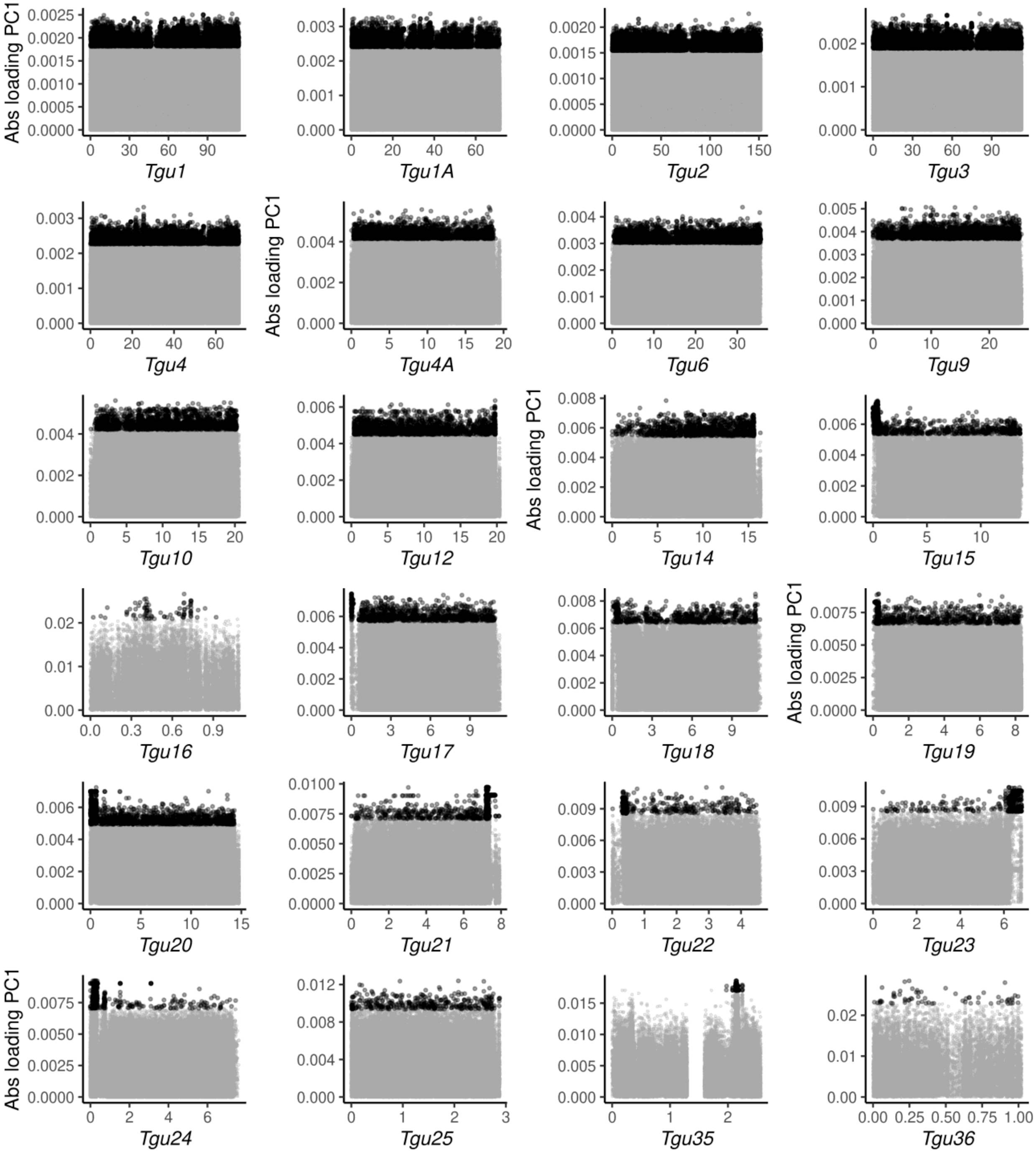

**Fig. S11.** PCA plots for chromosomes that show no signal of chromosomal inversions**.** For additional details see legend of **Fig. 1**.

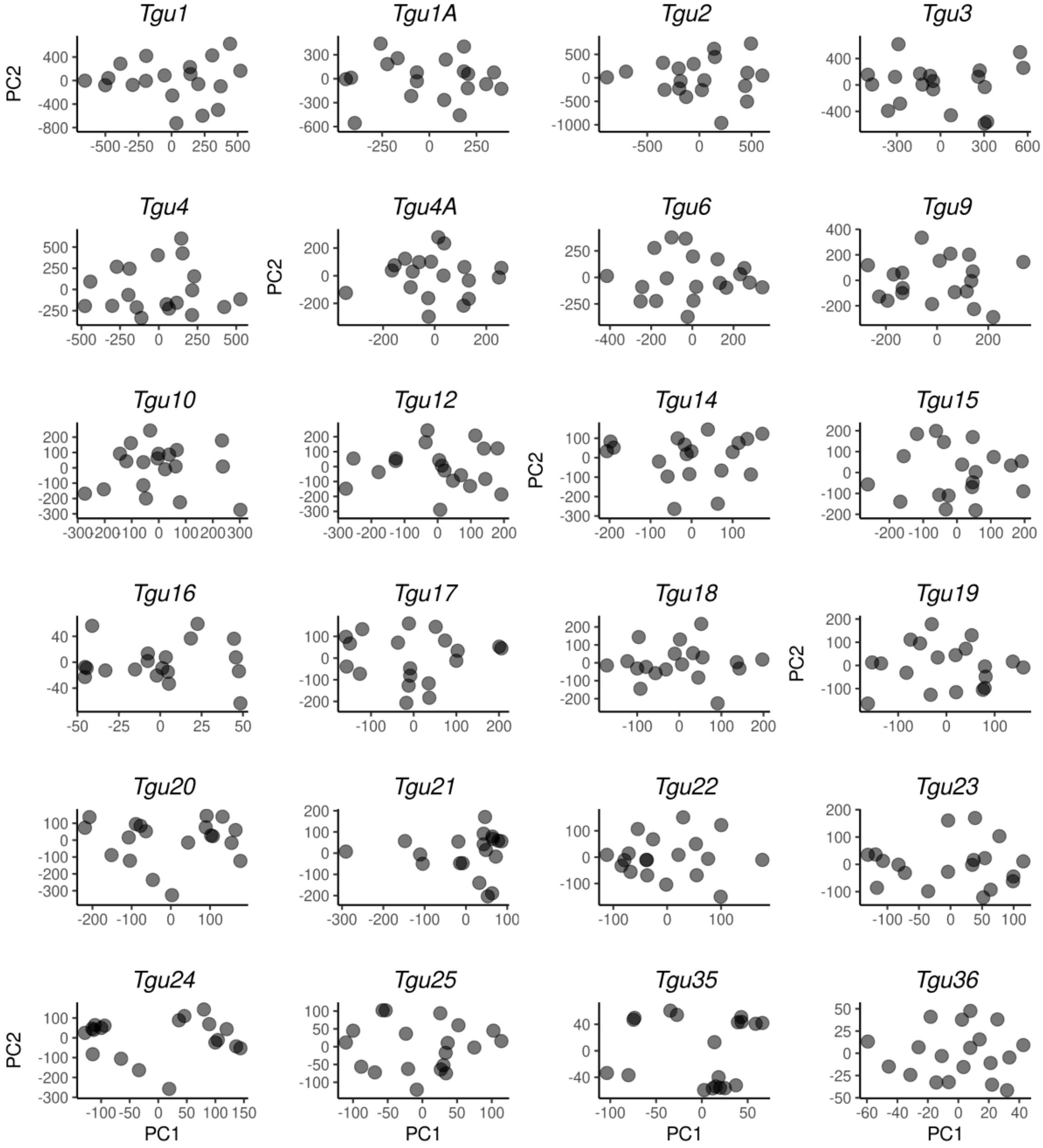

## References


Backström N, Forstmeier W, Schielzeth H, Mellenius H, Nam K, Bolund E, Webster MT, Ost T, Schneider M, Kempenaers B, et al. 2010. The recombination landscape of the zebra finch *Taeniopygia guttata* genome. *Genome Research* 20:485–495.

Hoffmann AA, Sgrò CM, Weeks AR. 2004. Chromosomal inversion polymorphisms and adaptation. *Trends in Ecology and Evolution* 19:482–488.

Hooper DM, Price TD. 2015. Rates of karyotypic evolution in Estrildid finches differ between island and continental clades. *Evolution* 69:890–903.

Ishii K, Charlesworth B. 1977. Associations between allozyme loci and gene arrangements due to hitch-hiking effects of new inversions. *Genet. Res., Camb* 30::93-106.

Kirkpatrick M. 2010. How and why chromosome inversions evolve. *PLoS Biology* 8.

Knief U, Hemmrich-Stanisak G, Wittig M, Franke A, Griffith SC, Kempenaers B, Forstmeier W. 2016. Fitness consequences of polymorphic inversions in the zebra finch genome. *Genome Biology* 17:199.

Knief U, Müller IA, Stryjewski KF, Metzler D, Sorenson MD, Wolf JBW. 2024. Evolution of chromosomal inversions across an avian radiation. *Molecular Biology and Evolution* 41:msae092.

Knief U, Schielzeth H, Ellegren H, Kempenaers B, Forstmeier W. 2015. A prezygotic transmission distorter acting equally in female and male zebra finches Taeniopygia guttata. *Molecular Ecology* 24:3846–3859.

Korunes KL, Noor MAF. 2018. Pervasive gene conversion in chromosomal inversion heterozygotes. *Molecular Ecology* 28:1302–1315.

Pei Y. 2022. Evolutionary genetics of reproductive performance in the zebra finch.

Pei Y, Forstmeier W, Ruiz-Ruano FJ, Mueller JC, Cabrero J, Camacho JPM, Alché JD, Franke A, Hoeppner M, Börno S, et al. 2022. Occasional paternal inheritance of the germline-restricted chromosome in songbirds. *Proc. Natl. Acad. Sci. U.S.A.* 119:e2103960119.

Rhie A, McCarthy SA, Fedrigo O, Damas J, Formenti G, Koren S, Uliano-Silva M, Chow W, Fungtammasan A, Kim J, et al. 2021. Towards complete and error-free genome assemblies of all vertebrate species. *Nature* 592:737–746.

Singhal S, Leffler EM, Sannareddy K, Turner I, Venn O, Hooper DM, Strand AI, Li Q, Raney B, Balakrishnan CN, et al. 2015. Stable recombination hotspots in birds. *Science* 350:928–932.

Stevison LS, Hoehn KB, Noor MAF. 2011. Effects of inversions on within- and between-species recombination and divergence. *Genome Biology and Evolution* 3:830–841.

Warren WC, Clayton DF, Ellegren H, Arnold AP, Hillier LW, Künstner A, Searle S, White S, Vilella AJ, Fairley S, et al. 2010. The genome of a songbird. *Nature* 464:757–762.
